# Heterozygous loss of *Zbtb38* leads to early embryonic lethality via the suppression of *Nanog* and *Sox2* expression

**DOI:** 10.1111/cpr.13215

**Published:** 2022-03-17

**Authors:** Miki Nishio, Takuya Matsuura, Shunya Hibi, Shiomi Ohta, Chio Oka, Noriaki Sasai, Yasumasa Ishida, Eishou Matsuda

**Affiliations:** ^1^ Functional Genomics and Medicine Nara Institute of Science and Technology Ikoma Japan; ^2^ Cosmo Bio Co., Ltd. Tokyo Japan; ^3^ Development Biomedical Science Nara Institute of Science and Technology Ikoma Japan

## Abstract

**Objectives:**

Mammalian DNA methyltransferases are essential to re‐establish global DNA methylation patterns during implantation, which is critical for transmitting epigenetic information to the next generation. In contrast, the significance of methyl‐CpG binding proteins (MBPs) that bind methylated CpG remains almost unknown at this stage. We previously demonstrated that Zbtb38 (also known as CIBZ)—a zinc finger type of MBP—is required for mouse embryonic stem (ES) cell proliferation by positively regulating Nanog expression. However, the physiological function of Zbtb38 in vivo remains unclear.

**Materials and Methods:**

This study used the Cre‐loxP system to generate conditional *Zbtb38* knockout mice. Cell proliferation and apoptosis were studied by immunofluorescence staining. Quantitative real‐time PCR, immunoblotting and immunofluorescence were performed to investigate the molecular mechanisms.

**Results:**

Germline loss of the *Zbtb38* single allele resulted in decreased epiblast cell proliferation and increased apoptosis shortly after implantation, leading to early embryonic lethality. Heterozygous loss of *Zbtb38* reduced the expression of *Nanog*, *Sox2*, and the genes responsible for epiblast proliferation, differentiation, and cell viability. Although this early lethal phenotype, Zbtb38 is dispensable for ES cell establishment and identity.

**Conclusions:**

These findings indicate that Zbtb38 is essential for early embryonic development via the suppression of *Nanog* and *Sox2* expression.

## Graphical abstract

Heterozygous loss of *Zbtb38* leads to aberrant epiblast cell proliferation and apoptosis shortly after implantation. Heterozygous loss of *Zbtb*38 reduced the expression of *Nanog* and *Sox2* in ICM and epiblast.

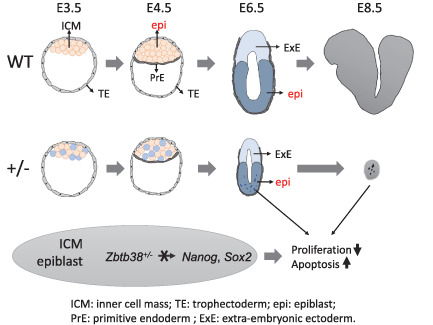

## 1 INTRODUCTION

Mammalian preimplantation development refers to the period from zygote to implantation of the blastocyst in the uterus.^1^ Mammalian preimplantation begins with zygotes that develop into two and four cells, followed by the formation of blastocysts that contain inner cell mass (ICM) and the trophectoderm (TE).^1^ Following implantation, ICM is further segregated into the epiblast and primitive endoderm (PrE), which develop into the embryo proper and yolk sac, respectively.^1^ Meanwhile, TE forms the extra‐embryonic ectoderm (ExE) and then gives rise to the placenta. In mice, implantation occurs at embryonic day (E) 4.5 (E4.5), early gastrulation occurs at E6.5, mid gastrulation at E7.5 and organogenesis from E8.5.^1^, ^2^ Previous evidence indicates that transcription factors play key roles in embryogenesis: epiblasts undergo proliferation via the core pluripotent transcription factors Nanog, Sox2 and Oct4, whereas PrE and TE undergo differentiation via Gata6 and Cdx2, respectively.^3^, ^4^

Pre‐ and peri‐implantation embryos undergo remarkable reprogramming through epigenetics, mainly including DNA methylation and histone modifications.^4^, ^5^, ^6^ DNA methylation is highly dynamic during mouse embryogenesis: early embryos give rise to extensive DNA demethylation from the zygote to the blastocyst, whereas their DNA methylation is globally re‐established during the implantation stage at E4.5–E6.5.^7^, ^8^ Notably, the DNA methylation level in ICM of the blastocyst is much higher than that in TE.^9^, ^10^ This difference becomes significantly apparent by E6.5, when the epiblast performs most of the DNA methylation as compared to the lower methylation state of the ExE.^11^ DNA methylation is mediated by a family of conserved DNA methyltransferases (Dnmts) and MBPs. Dnmt3a and Dnmt3b are responsible for de novo DNA methylation, whereas Dnmt1 maintains methylation patterns after DNA replication.^12^, ^13^ In contrast, MBPs bind to the methylated CpG and generally repress gene expression, but they are also associated with gene activation.^13^ Accumulating evidence has demonstrated that DNA methylation plays key roles in mammalian development and is involved in various biological processes, including gene regulation, transposon silencing, lineage specification, genomic imprinting, and X chromosome inactivation.^7^, ^13^, ^14^, ^15^ In previous studies, Dnmt3a/Dnmt3b double knockout (KO) mice and Dnmt1 KO mice resulted in embryonic lethality around E9.5,^16^, ^17^ indicating that both de novo DNA methylation and its maintenance are critical for early embryo viability.

MBPs are classified into two structural families: the methyl‐CpG‐binding domain family (Mecp2, Mbd1, Mbd2 and Mbd4), the zinc finger family (Kaiso/Zbtb33, Zbtb4, Zbtb38 and Zfp57).^14^, ^18^, ^19^ To date, genetic studies on the MBP single gene KO mice except for *Zbtb38*, double and triple MBP (*Mecp2*, *Mbd2* and *kaiso*) gene KO mice have demonstrated that all of these are dispensable for early embryogenesis.^20^, ^21^, ^22^, ^23^, ^24^, ^25^, ^26^ Thus, extensive functional redundancy could not explain the discrepancy between these MBPs in mouse embryogenesis. In addition, Zfp57 binds to certain imprinting control regions, and *Zfp57* homozygous KO (*Zfp57* ^*−/−*^) led to late embryonic (E14.5~) lethality due to defects in the maintenance of both maternal and paternal imprints.^27^ So far, the significance of MBPs at the peri‐implantation stage remains unclear despite Dnmts playing key roles at this stage.

We previously showed that Zbtb38/CIBZ binds to methylated DNA via zinc fingers to repress or activate transcription via the BTB and spacer domain, respectively.^28^, ^29^ We also demonstrated that Zbtb38 promotes ES cell proliferation, inhibits ES cell differentiation toward the mesodermal lineage, and suppresses apoptosis in murine cells.^30^, ^31^, ^32^ Intriguingly, Zbtb38 loss in ES cells decreased Nanog expression and, consequently, abrogated ES cell proliferation by inhibiting the G1/S transition.^30^ Nanog is one of the first markers to be restricted within the epiblast, followed by Sox2 and Oct4, all of which are capable of regulating self‐renewal and pluripotency in ES cells and epiblast cells.^33^ Nanog is rapidly downregulated after the blastocyst stage but is expressed again in the posterior epiblast from E6.5 onwards,^34^, ^35^ and Sox2 is localized to the prospective neuroectoderm in the anterior epiblast.^36^

Besides Zbtb38's cellular functions in murine cells, it also has important physiological roles in humans. In humans, several lines of evidence indicate that the expression level of ZBTB38 is important for its normal physiological functions, and its misregulation leads to numerous diseases.^37^, ^38^, ^39^, ^40^, ^41^, ^42^ Genome‐wide association studies in humans indicated that the strongest association of human height with the SNP is the ZBTB38 gene, and this SNP in which the A allele of rs6763931 in the region has the most significant correlation with its expression in blood.^37^ Moreover, expressions of ZBTB38 are associated with the development of cancers^38^, ^39^, ^40^ and neurodegenerative disease.^41^, ^42^

The above findings show that Zbtb38 expression is essential not only in vitro but also in vivo. In this study, we generated *Zbtb38* heterozygous KO (*Zbtb38* ^*+/−*^) mice using conventional and Cre‐loxP‐based conditional KO (cKO) approaches to reveal the physiological role of Zbtb38 in mice. Our results from the two different strategies demonstrated that loss of the *Zbtb38* single allele led to embryonic developmental failure. We presented the data indicating that loss of Zbtb38 decreased the expression of *Nanog* and *Sox2*, both of which are critical for epiblast proliferation and differentiation.

## 2 MATERIALS AND METHODS

### 2.1 Generation of *Zbtb38 fl/+* ES cells‐ and *Zbtb38* ∆*fl/+* mice

The targeting vector was purchased from European Conditional Mouse Mutagenesis Program (EUCOMM, ID: MAE‐2331), encompassing a 25‐kilobase (kb) DNA fragment including 10 kb of *Zbtb38* homologous sequence in which the 5′ and 3′ arms of homology are 6.2 and 4.1 kb, respectively (Figure S2A). The recombinant allele containing an FRT‐flanked LacZ and neo cassettes was linearized with *AsiS*I and was electroporated into 129‐derived ES cells. After electroporation, ES cell clones were grown in the presence of 500 μg/ml geneticin and isolated after culturing for 8–12 days on mitomycin C‐treated SNL‐STO cells as described previously.^31^ The correct integration of the targeting construct and homologous recombination was confirmed by genomic PCR (gPCR) and restriction enzyme digestion (Figure S2B), and sequencing, respectively. ES cells were cultured in ES medium (DMEM, 15% foetal bovine serum, 2 mM L‐glutamine, 100 μM nonessential amino acids, 1% penicillin and streptomycin, and 0.1 mM β‐mercaptoethanol) supplemented with 1000 IU/ml recombinant murine leukaemia inhibitory factor (LIF, NU‐0012, Nacalai Tesque) as described previously.^31^

The proper recombinant ES clone was injected into C57BL/6J blastocysts with subsequent transplantation into pseudo‐pregnant female mice. Chimeric males were selected based on coat colour and subsequently crossed with C57BL/6J female mice to generate the *Zbtb38 fl‐neo/+* mice. Male *fl‐neo/+* mice were crossed to the C57BL/6J females to generate F1 offspring. The *Zbtb38 fl‐neo/+* mice were crossed with FLP^43^ mice to remove the FRT‐flanked splice acceptor sites to generate *Zbtb38 fl/+* mice. The *Zbtb38 fl/+* mice were crossed with a CAG‐Cre strain that ubiquitously expresses Cre recombinase to obtain the *Zbtb38 ∆fl/+* mice. *Zbtb38 fl/+* mice were backcrossed with C57BL/6J at least for eight generations. A single male was paired with one or two females, which was plug‐checked and weighed daily. The embryonic days were counted starting at E0.5 on the day the vaginal plug was detected. C57BL/6J mice were purchased from CLEA Japan. Gt (ROSA)26Sortm1(FLP1) Dym (Rosa‐Flp) mice were obtained from Jackson Laboratory. CAG‐Cre mice were provided by Dr. Masaru Okabe (Osaka University). All mice were approved by the Animal Care Committee of Nara Institute of Science and Technology and conducted in accordance with guidelines that were established by the Science Council of Japan.

### 2.2 Genotyping

Genomic DNA was prepared from the ear punching with Gentra Puregene Cell Kit (Qiagen), or from embryos at various developmental stages with Gentra Puregene Cell Kit or AllPrep DNA/RNA FFPE Kit (Qiagen). All experiments were conducted according to the manufacturer's instructions. Approximately 1–100 ng of DNA was used for genotyping by PCR analysis. Information of primers was shown in Table S3.

### 2.3 Two‐cell and blastocyst collection and ES cell establishment

Isolation and establishment of blastocysts was performed according to the protocol described^44^ with slight modification. Briefly, two‐cell or blastocyst was obtained from natural mating or superovulation. For superovulation, female mice (4–8 weeks old) were intraperitoneally injected with 7.5 IU pregnant mare's serum gonadotrophin, followed 48 h later by an intraperitoneal injection of 7.5 IU of human chorionic gonadotrophin (hCG), both were purchased from ASKA Pharmaceutical Co., Ltd. Female mice were mated with males immediately after hCG injection. Approximately 44 h after hCG injection, two‐cell embryos were flushed from oviduct at E1.5 and cultured in M16 medium (M7292, Sigma‐Aldrich) covered with mineral oil until they reached the blastocyst stage. For blastocysts isolation, E3.5 embryos were flushed from uterine horns, and cultured with M16 medium under a 5% CO2, 37°C incubator.

For generating ES cells, individual late‐cavitating blastocysts of similar morphology from each experimental group were treated with Tyrode's solution (T1788, Sigma‐Aldrich) to remove the zona pellucida, then transferred onto the SNL‐STO feeder cells. Blastocysts were cultured in ES medium with 1000 IU/ml LIF and the differentiation inhibitors 3i (3 μM CHIR99021, 1 μM PD0325901 and 10 μM SB431542, all were purchased from Selleck Chemicals). After 5 days of culture, ICM outgrowths were individually collected, dissociated with trypsin‐EDTA, and transferred the cells to 24‐wells plated on feeder layers. The resulting undifferentiated colonies were further propagated in the same medium. Putative ES cell lines were passaged several times in ES medium containing the differentiation inhibitors 3i. ES cells were characterized by their morphology as captured by Nikon Diaphot 300 inverted microscope, and by an Alkaline phosphatase assay. Genomic DNA extracted from the ES cells was genotyped by the gPCR. PCR genotyping with primers a ~ d. WT (primers a–b), *fl* (primers a–b) and ∆*fl* (primers c–d) alleles produced 337‐bp, 541‐bp and 362‐bp bands, respectively. Primers were listed in Table S3.

### 2.4 Blastocyst outgrowth assay

The blastocyst outgrowth assay was performed as described,^45^ with slight modifications. Briefly, blastocysts with or without the zona pellucida were plated into 0.1% gelatin‐treated 24‐well plates and cultured in ES medium lacking LIF. Embryos were imaged daily with a NikonDiaphot 300 inverted microscope. On day 4 (4 DIV), embryos were fixed with 4% paraformaldehyde, imaged, then the DNA was extracted and genotyped by PCR. Areas from the four DIV ICM and TE outgrowths were calculated from images using ImageJ software, and the ICM ratio (defined as ICM area/TE area) was calculated using Excel.

### 2.5 Alkaline phosphatase assay

Alkaline phosphatase activity of undifferentiated ES cells was detected using the Alkaline Phosphatase Staining Kit II (00‐0055, Stemgent), according to manufacturer's instructions.

### 2.6 Embryo dissection and histological analysis

Morphological and histological analyses of embryos were collected at various times of gestation. For histological analysis, embryos were rinsed with ice‐cold PBS and fixed with 4% paraformaldehyde at room temperature for 10–30 min, dehydrated through graded alcohols, and embedded in paraffin. Embryos were sectioned at 5 μm thickness and stained with haematoxylin and eosin. Sectioned embryos were photographed using a Nikon E800M inverted microscope. For immunofluorescent analysis, paraffin sections were rehydrated, immunostained with cleaved Caspase‐3 antibody (9664, Cell Signalling Technology), Ki‐67 antibody (12202, Cell Signalling Technology). Antigen retrieval was performed according to the individual antibody instructions. CF488A Donkey anti‐Rabbit IgG (20015, Biotium) and CF488A Donkey anti‐Mouse IgG (20,014, Biotium) were used as the secondary antibody. Nuclei were counterstained with 1 μg/ml Propidium Iodide (P4170, Sigma‐Aldrich), washed and mounted.

Terminal deoxynucleotidyl transferase (TdT) mediated dUTP nick end‐labeling (TUNEL) assay was performed using MEBSTAIN Apoptosis TUNEL Kit Direct (8445, MBL). Briefly, paraffin‐embedded sections were deparaffinized, DNA nick end‐labelled, and counterstained with 1 μg/ml Propidium Iodide and mounted. The slides were analysed using a TCS SP8 confocal microscope (Leica).

### 2.7 BrdU incorporation assay

For 5‐bromo‐2′‐deoxyuridine (BrdU; Sigma‐Aldrich) incorporation, pregnant female mice at specific times were injected intraperitoneally with BrdU (50 μg/g of body weight). The mice were sacrificed by cervical dislocation after 2 h injection. For histological analysis, embryos and tissues were fixed in 4% paraformaldehyde overnight at 4°C and embedded in paraffin wax for sectioning. Five‐micrometre sections were deparaffinized, and incubated with anti‐BrdU antibody (66241‐1, Proteintech) after heat antigen retrieval and denaturation according to the manufacturer's instructions. Anti‐Mouse CF488A was used as the secondary antibody. After staining, the slides were counterstained with 1 μg/ml propidium iodide. Blastocysts or blastocysts from outgrowth were treated with 20 μM BrdU for 2–4 h before 4% paraformaldehyde fixed and immunostained.

### 2.8 Whole‐Mount Immunohistochemistry for embryos

Whole‐Mount Immunohistochemistry was performed as described^46^ with a slight modification. Briefly, embryos (blastocysts, blastocyst from outgrowth, E6.5–E7.5 embryos) were washed three times with cold‐PBS, and fixed with methanol/DMSO (4:1) at 4° C overnight. Embryos were treated with methanol/DMSO/H_2_O_2_ for 3 h, and rehydrated with 75%, 50% and 25% methanol. After 30 min of incubation with 0.3% Triton X‐100 in PBS, embryos were blocked with the washing buffer (0.2% Tween 20 in PBS) supplemented with 10% goat serum at room temperature for 120 min or overnight at 4° C. After washing several times with the washing buffer, embryos were incubated with anti‐Zbtb38 antibody,^29^ anti‐Nanog (560259, BD Pharmingen), anti‐Sox2 (S1451, Sigma‐Aldrich), anti‐Oct4 (sc‐5279, Santa Cruz Biotechnology) for 120 min, washed with washing buffer, and then incubated with secondary antibody for 60 min. Secondary antibodies used were Alexa Fluor 594 goat anti‐Mouse IgG (8890, Cell Signalling Technology) and Alexa Fluor 488 goat anti‐Rabbit IgG (A32731, Thermo Fisher Scientific). Nuclei were counterstained with 4′,6‐diamidino‐2‐phenylindole (DAPI). After washing with washing buffer, the signal was viewed under a confocal laser‐scanning microscope (TCS SP8, Leica). All procedures were performed in a six‐well plate except for the experiment with outgrowth embryos, which was performed in a four‐well plate. Quantitative analysis of immunofluorescence was performed using ImageJ.

### 2.9 RNA extraction and quantitative real‐time PCR

Total RNA was extracted using Sepasol (Nacalai Tesque) for beyond E9.5 embryo, AllPrep DNA/RNA FFPE Kit (Qiagen) for paraffin section, PicoPure™ RNA Isolation Kit (Thermo Fisher Scientific) for blastocyst‐E8.5 embryos, according to the manufacturer's instructions. Approximately 5 ng total RNA from blastocysts and E6.5–E7.5 embryos, and 50–500 ng total RNA from E8.5 beyond embryos were used to synthesize cDNA. cDNA was synthesized using ReverTra Ace qPCR RT Master Mix with gDNA Remover (TOYOBO). Quantitative real‐time PCR (qRT‐PCR) was performed using a LightCycler® 96 System (Roche Diagnostics) with the Thunderbird SYBR Green PCR Mix (TOYOBO), following the manufacturer's instructions as described.^31^ cDNA samples were analysed in triplicate on 96‐well optical PCR plates (Roche Diagnostics). *GAPDH* or *TBP* was used as the reference gene and all analyses were performed using the 2‐Deltadelta Ct method with Roche LightCycler 96 system software. Primer sequences are listed in Table S2.

### 2.10 Western blotting

Western blotting was performed as described previously.^28^ Briefly, protein lysates were prepared in RIPA buffer supplemented with complete protease inhibitor (Roche Diagnostics). Proteins were then separated on 6%–15% SDS‐PAGE, transferred onto PVDF membranes, and probed with anti‐Zbtb38,^29^ anti‐Oct3/4 (MAB1759, R&D Systems), anti‐Sox2 (S1451, Sigma‐Aldrich), anti‐Nanog (AB5731, Millipore), anti‐α‐tubulin (T6199, Sigma‐Aldrich) antibodies. HRP‐conjugated anti‐mouse (7076, Cell Signalling Technology) or anti‐rabbit IgG (7074, Cell Signalling Technology) were used as secondary antibodies. Quantification was conducted by using GelQuant.NET software provided by biochemlabsolutions.com.

### 2.11 Statistical analysis

Unless stated otherwise, data are given as means ± standard deviation (SD). Each experiment included at least three independent samples and was repeated at least three times. Statistical analyses were performed with GraphPad Prism 7.0 software using two‐tailed unpaired Student's *t*‐test, and differences were considered significant when *p < 0.05, **p < 0.005. ‘n.s.’ indicates no significance (*p* > 0.05).

## 3 RESULTS

### 3.1 *Zbtb38* expression is up‐regulated during embryogenesis, and heterozygous loss of *Zbtb38* results in embryonic lethality

**FIGURE 1:**
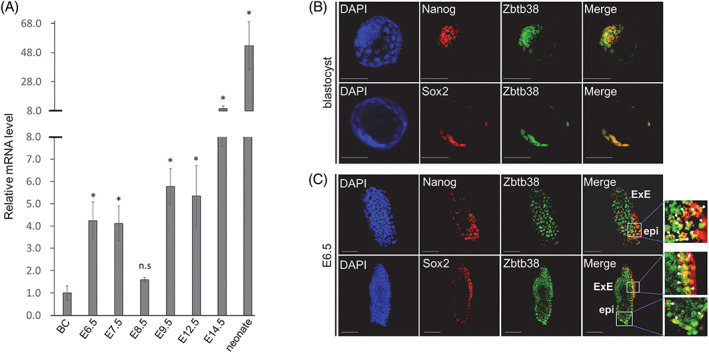
Expression of Zbtb38 during mouse embryonic development. (A) Results of the fold change of qRT–PCR for *Zbtb38* expression during mouse embryonic development. The graph shows the fold change relative to blastocyst, which is denoted by 1. Embryos from the indicated stages were carefully isolated from the uterus of C57BL/6J mice intercrosses. The TATA‐binding protein (TBP) gene was used as an internal control, and the levels of transcripts were normalized against the TBP gene. Data are representative of three independent replicates measured in triplicates, and error bars indicate ± S.D. *p < 0.05. ‘n.s.’ indicates no significance. (B and C) Whole‐mount immunofluorescence and confocal microscopy for blastocyst (B) and E6.5 (C). Expressions of the indicated proteins were detected using anti‐ Zbtb38, anti‐Nanog and anti‐Sox2 antibodies. Cell nuclei were counterstained with DAPI. White arrows indicate regions of colocalization. Scale bar denotes 50 μm (1B) or 100 μm (1C). epi, epiblast; ExE, extraembryonic ectoderm

To gain insights into the role of Zbtb38 in vivo, we quantified the expression levels of *Zbtb38* during embryogenesis in wild‐type (WT, C57BL/6J) embryos using qRT‐PCR. The results showed that *Zbtb38* mRNA was expressed at a detectable level at the blastocyst stage (Figure 1A), which is consistent with the results of our previous study that showed high expression of *Zbtb38* in ES cells.^31^ In the later stages, *Zbtb38* transcripts were increased between E6.5 and E7.5 by four‐fold, but decreased at E8.5, and elevated again at E9.5, and the increase lasted until the neonatal stage (Figure 1A). In addition, immunofluorescence analysis using the Zbtb38 antibody showed that Zbtb38 was predominantly expressed in the ICM of blastocysts and was largely colocalized with the ICM markers—Nanog, Sox2 (Figure 1B), and Oct4 (Figure S1A). Zbtb38 staining was also weakly detected in TE. At E6.5, Zbtb38 was found to be largely expressed in the epiblast and was partially colocalized with epiblast markers of Nanog, Sox2 (Figure 1C), and Oct4 (Figure S1B). Additionally, Zbtb38 colocalized with Sox2 at ExE (Figure 1C). The characteristic expression pattern of Zbtb38 implies that it is associated with early embryonic development with unique features.

**FIGURE 2:**
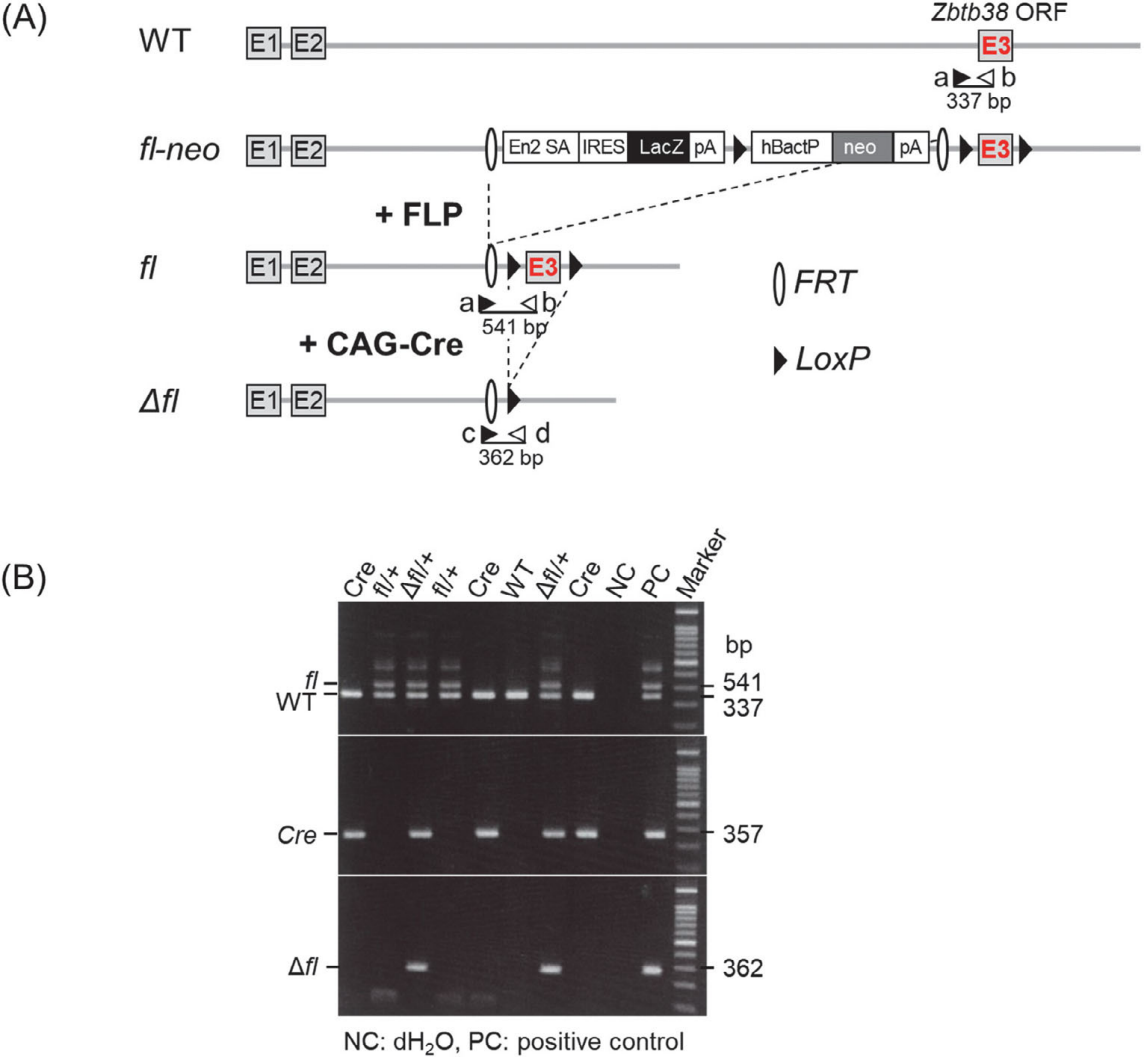
Generation of the *Zbtb38* heterozygous KO mice. (A) Schematic diagram of gene targeting strategy. Crossing the *fl‐neo* allele to a mouse line expressing FLP recombinase removes the lacZ and neo cassettes, resulting in a conditional‐ready allele (*fl*). When the *fl* allele is crossed with a mouse strain expressing CAG‐Cre recombinase, exon 3 is deleted, resulting in a null allele (∆*fl*). Exons are shown as empty boxes and marked by a number inside. LoxP sites (black triangles) and FRT sites (empty semicircles) are shown. En2 SA, mouse En2 splicing acceptor; FLP, flp recombinase; hBactP, human b‐actin promoter; IRES, internal ribosome entry site; neo, neomycin‐resistant gene; pA, poly(A) signal. The position of the primers (a‐d) used for genotyping is shown with arrows, and the expected sizes (bp) of genomic PCR are shown under the individual primer pairs. (B) PCR genotyping of paraffin section of E8.5 embryos isolated from the *Zbtb38 fl/+* mice and the CAG‐Cre mice intercrosses. Representative PCR genotyping with primers a ~ d, WT, *fl* and *∆fl* alleles produced a 337‐bp, 541‐bp and 362‐bp bands, respectively. Cre genotyping primer produces a 357 bp band

To investigate the physiological role of Zbtb38 during embryogenesis, the conventional KO strategy was used to establish two independent *Zbtb38* ^*+/−*^ ES cell clones that were able to maintain pluripotency and self‐renewal, as described previously.^30^ However, when the *Zbtb38* ^*+/−*^ ES cells were microinjected into blastocysts to generate chimeric mice, no chimeric neonate was obtained. This finding raised the possibility that these heterozygous ES cell‐derived chimera embryos did not survive in the uterus. To test this possibility and determine the timing of abnormality, we generated *Zbtb38* cKO mice using the Cre‐LoxP system. Two approaches were carried out to generate the *Zbtb38* single null alleles (Figure S3A–D). The first was crossing the *Zbtb38 flox (fl)‐neo/+* mice with CAG‐Cre mice expressing Cre recombinase ubiquitously under the CAG promoter.^47^ This intercross resulted in the removal of the *Zbtb38* exon3, which encodes a single transcript that produces the entire Zbtb38 protein, leading to the germline deletion of the *Zbtb38* single allele (*∆f‐neo*, Figure S4A). To rule out the possibility that the remaining lacZ gene has an unexpected effect, the second approach, which requires two steps, was also performed: (A) The *Zbtb38 fl‐neo/+* mice were crossed with FLP transgenic mice,^43^ leading to deletion of the *lacZ* and *neomycin* cassettes (*fl*, Figure 2A). (B) The *Zbtb38 fl/+* mice were subsequently crossed with CAG‐Cre mice, resulting in the germline deletion of the *Zbtb38* single allele (*∆fl*, Figure 2A). Genomic PCR (gPCR) analysis revealed successful deletion of the *Zbtb38* exon3 and corresponding cassettes in both methods (Figure 2B; Figure S4B), indicating that the loxP and FRT sites are functional in vivo.

We found that either the *Zbtb38 fl‐neo/+* or the *Zbtb38 fl/+* mice were viable, fertile, and morphologically indistinguishable from their WT or CAG‐Cre littermates for at least 18 months of breeding (data not shown). Crossing the *Zbtb38 fl/+* with the CAG‐Cre mice generated Mendelian ratios of WT, *fl/+*, and CAG‐Cre neonates, but the *Zbtb38 ∆fl/+* was not identified (Table 1). Likewise, out of 96 mice born from the *fl‐neo/+* and CAG‐Cre intercrosses, *Zbtb38 ∆fl‐neo/+* neonates were not found, whereas the remaining genotypes displayed the expected Mendelian ratios (Table S1). Together, these results demonstrate that the loss of a single *Zbtb38* allele leads to embryonic lethality.

**TABLE 1:** Genotype analysis of offspring from the *Zbtb38 fl/+* and CAG‐Cre intercrosses

Stage	Total	WT	*fl*/+	Cre	Δ*fl*/+
Newborn	102	31	36	35	0
E11.5	30	9	13	8	0
E9.5	65	18	23	20	4 (4)^a^
E8.5	85	21	25	24	15 (15)^b^
E7.5	85	17	23	21	24 (24)^b^
E6.5	66	14	18	21	13 (9)^b^

^a^ Absorbed.

^b^ Abnormal.

### 3.2 Heterozygous loss of *Zbtb38* leads to early post‐implantation defects

**FIGURE 3:**
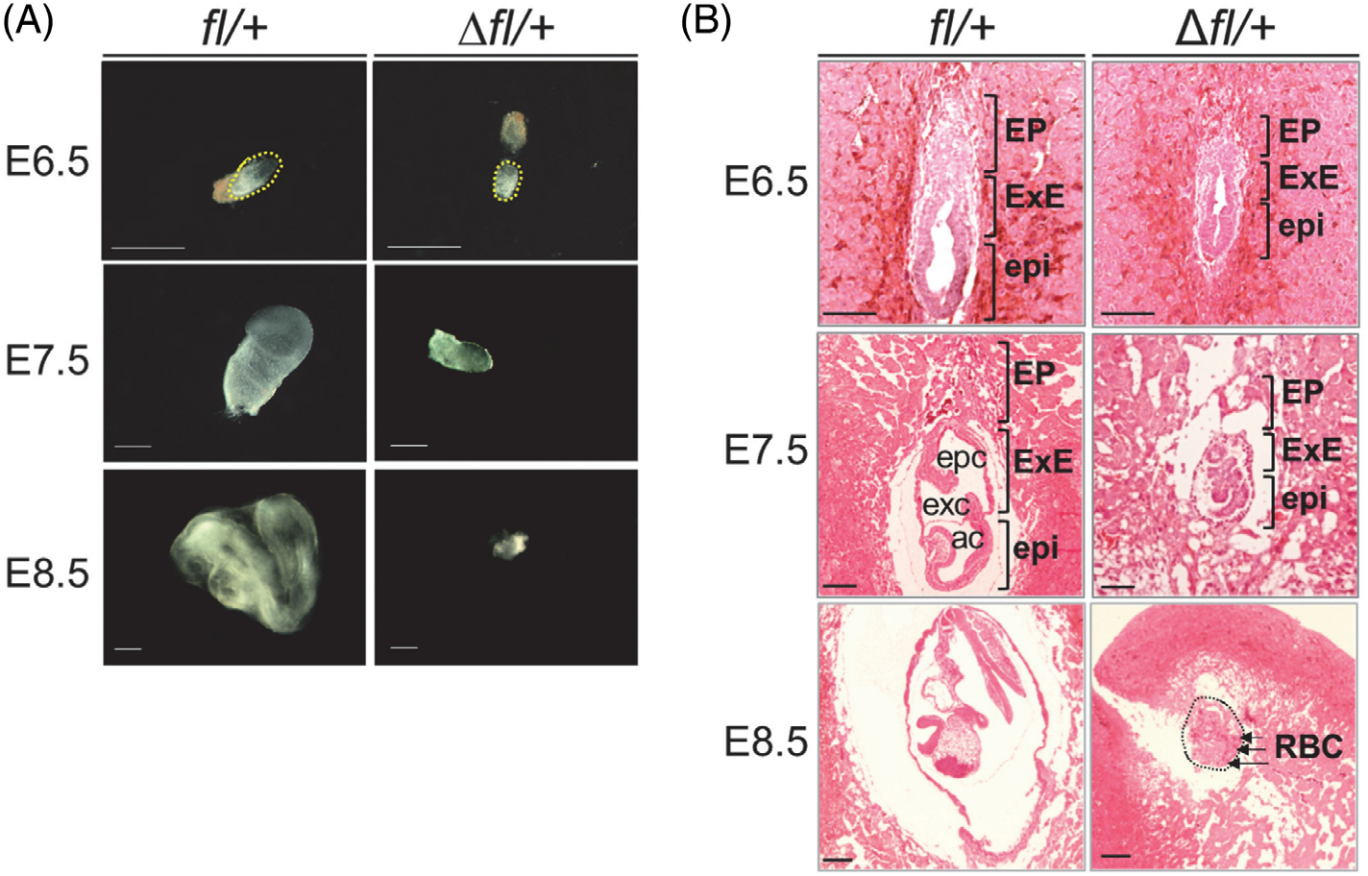
Heterozygous loss of *Zbtb38* impairs embryonic development. (A and B) Heterozygous loss of *Zbtb38* leads to abnormal embryo development during E6.5–E8.5. (A) Bright‐field images of representative *Zbtb38 fl/+* and Δ*fl/+* embryos at the indicated stages of gestation. Scale bar, 500‐μm. (B) H&E staining for sagittal sections of *Zbtb38* ∆*fl/+* and *fl/+* embryos at the indicated stages. ac, amniotic cavity; EP, ectoplacental corn; epc, ectoplacental cavity; epi, epiblast; exc, exocoelomic cavity; ExE, extraembryonic ectoderm; RBC, red blood cells. Scale Bar: 50 μm

To determine the timing of embryonic lethality, we examined the embryonic morphology at different gestation stages. In an analysis of the littermates from the *Zbtb38 fl/+* and CAG‐Cre intercross, the expected Mendelian ratios of the four expected genotypes were found to be E6.5–E8.5 (Table 1). In contrast, beyond E9.5, no Δ*fl/+* embryos were identified, whereas the other genotypes were morphologically normal with the expected Mendelian ratio (Table 1). Likewise, *∆fl‐neo/+* embryos were not found beyond E9.5, whereas the other genotypes displayed the expected Mendelian frequency (Table S1). These results indicated that *Zbtb38* ^*+/−*^ embryos died around E9.5. Approximately, 70% (*n* = 9 of 13) of the Δ*fl/+* embryos were smaller in size than their littermate controls at E6.5 (Figure 3A). At E7.5, the size difference between all Δ*fl/+* embryos (n = 24 of 24) and controls was more obvious. At E8.5, the Δ*fl/+* mice were considerably smaller in size and abnormal in morphology compared with the controls (Figure 3A). To further analyse the *Zbtb38* heterozygous phenotype, we examined histological sections of embryos using haematoxylin and eosin staining. Compared with littermate controls at E6.5, Δ*fl/+* embryos showed similar morphological characteristics; however, their epiblasts and ExE were smaller and less organized (Figure 3B). At E7.5, control embryos increased in size and further progressed to form the amniotic, exocoelomic, and ectoplacental cavities. In comparison, all *Zbtb38* ∆*f/+* embryos displayed compact, severely underdeveloped cavities (Figure 3B). At E8.5, all *Zbtb38* mutant embryos were significantly smaller than the other genotypes of embryos and were absent from typical organogenesis, which was usually observed in control embryos. Moreover, the embryos exhibited massive red blood cell infiltration (Figure 3B). Collectively, these results indicate that heterozygous loss of *Zbtb38* leads to abnormal embryo development starting from E6.5, progressing at E7.5–E8.5, and leading to embryonic lethality at E9.5.

### 3.3 Heterozygous loss of *Zbtb38* results in decreased proliferation and increased apoptosis in embryos

**FIGURE 4:**
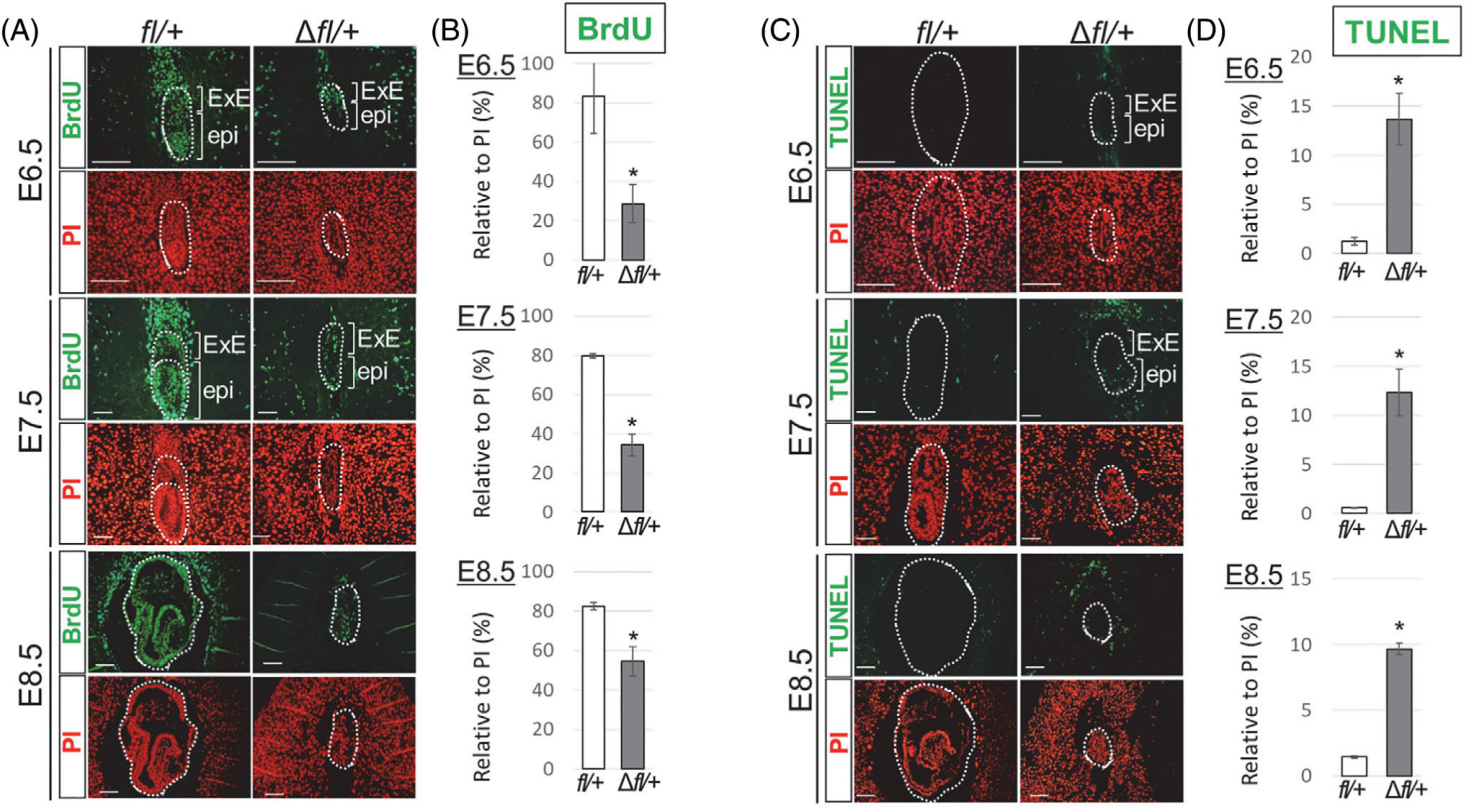
Evaluation of proliferating and apoptotic cells in E6.5–E8.5 embryos. (A and B) Heterozygous loss of *Zbtb38* inhibits epiblast cell proliferation of E6.5–E8.5 embryos. (A) Immunofluorescence analysis of paraffin‐embedded sections of controls and *Zbtb38* ∆*fl/+* embryos at E6.5–E8.5. Two consecutive paraffin‐embedded sections were taken for performing immunostaining with anti‐BrdU antibody (green), and nuclei were counterstained with PI (red). epi, epiblast; ExE, extraembryonic ectoderm. Scale Bar: 50 μm. (B) Quantitative analysis of the number of labelled BrdU cells relative to the total number of PI‐positive nuclei from the indicated umbers of embryos at E6.5 (*fl/+*: *n* = 9; ∆*fl/+*: *n* = 8), E7.5 (*fl/+*: *n* = 11; ∆*fl/+*: *n* = 9), and E8.5 (*fl/+*: *n* = 6; ∆*fl/+*: *n* = 5). Error bars represent ± S.E.M. *p < 0.05. (C and D) Heterozygous loss of *Zbtb38* induces apoptosis of E6.5–E8.5 embryos. (C) A TUNEL assay was performed on paraffin‐embedded sagittal sections from E6.5 embryos onwards. TUNEL‐positive cells are shown in green, and nuclei were counterstained with PI (red). Scale Bar: 50 μm. (D) Quantitative analysis of the number of TUNEL‐positive cells relative to the total number of PI‐positive nuclei from the indicated umbers of embryos at E6.5 (*fl/+*: *n* = 9; ∆*fl/+*: *n* = 7), E7.5 (*fl/+*: *n* = 11; ∆*fl/+*: *n* = 8), and E8.5 (*fl/+*: *n* = 7; ∆*fl/+*: *n* = 5). Error bars indicate ± S.E.M. *p < 0.05

Because heterozygous mice exhibited a smaller body size, we reasoned that cell proliferation was affected in these embryos. To investigate cell proliferation in the embryos, we performed BrdU incorporation and immunohistochemical analysis of Ki67 in embryos prepared with paraffin sections. In these assays, the cells in the S phase are labelled with BrdU, whereas the cells in the G1, S, G2, and M phases are detected with Ki67. As observed in Figure 4A,B, cells of the control embryos displayed considerable BrdU incorporation in both the epiblast and ExE at E6.5–E8.5 (>80%). In contrast, in the *Zbtb38* ^*+/−*^ embryo, BrdU‐positive cells of epiblast and ExE were dramatically reduced at E6.5 (dropped by 65%), E7.5 (dropped by 57%), and E8.5 (dropped by 34%). Notably, the decrease in BrdU incorporation in the *Zbtb38* ^*+/−*^ embryos was predominantly observed in the epiblast than in the ExE (Figure 4A,B). In comparison, both the *Zbtb38* ∆*f/+* embryos and control embryos exhibited a high percentage of ki67‐positive cells at E6.5‐E8.5 (>80%), and no significant difference was observed between them (Figure S5A,B). Consistent with these results, our previous analysis showed that loss of Zbtb38 functions by gene KO or by siRNA‐mediated knockdown in the ES cells inhibited BrdU incorporation, impaired G1 to S transition, and therefore, suppressed proliferation.^30^ Taken together, these findings demonstrate that heterozygous loss of *Zbtb38* inhibits G1 to S transition of epiblast cells, and consequently, leads to developmental failure of the embryo.

To explore the fate of the decreased proliferating cells, we speculated that these cells undergo apoptosis. Therefore, we conducted a TUNEL assay, which is the most commonly employed method to detect the fragmented DNA characteristic of apoptotic cells, in paraffin‐embedded sections of embryos. As shown in Figure 4C,D, dramatically increased TUNEL‐positive nuclei were detected in the *Zbtb38* ∆*f/+* embryos (>10‐fold), particularly in the epiblast at E6.5–E7.5, whereas only few such cells were detected in the control embryos at E6.5–E8.5 (<2%). Moreover, cleaved Caspase‐3 (cCasp3), another apoptosis marker, was also examined. Similar to the TUNEL analysis, an increased number of cCasp3‐positive cells was detected (>10‐fold), especially in the *Zbtb38* ^*+/−*^ epiblast at E6.5‐E8.5 (Figure S6A,B), whereas positive cells were rarely observed in control embryos. Together, these findings indicate that heterozygous loss of *Zbtb38* induces apoptosis during early embryonic development and aggravates developmental defects.

### 3.4 Heterozygous loss of *Zbtb38* does not affect the development of 2C to the blastocyst stage but inhibits blastocyst outgrowth

**FIGURE 5:**
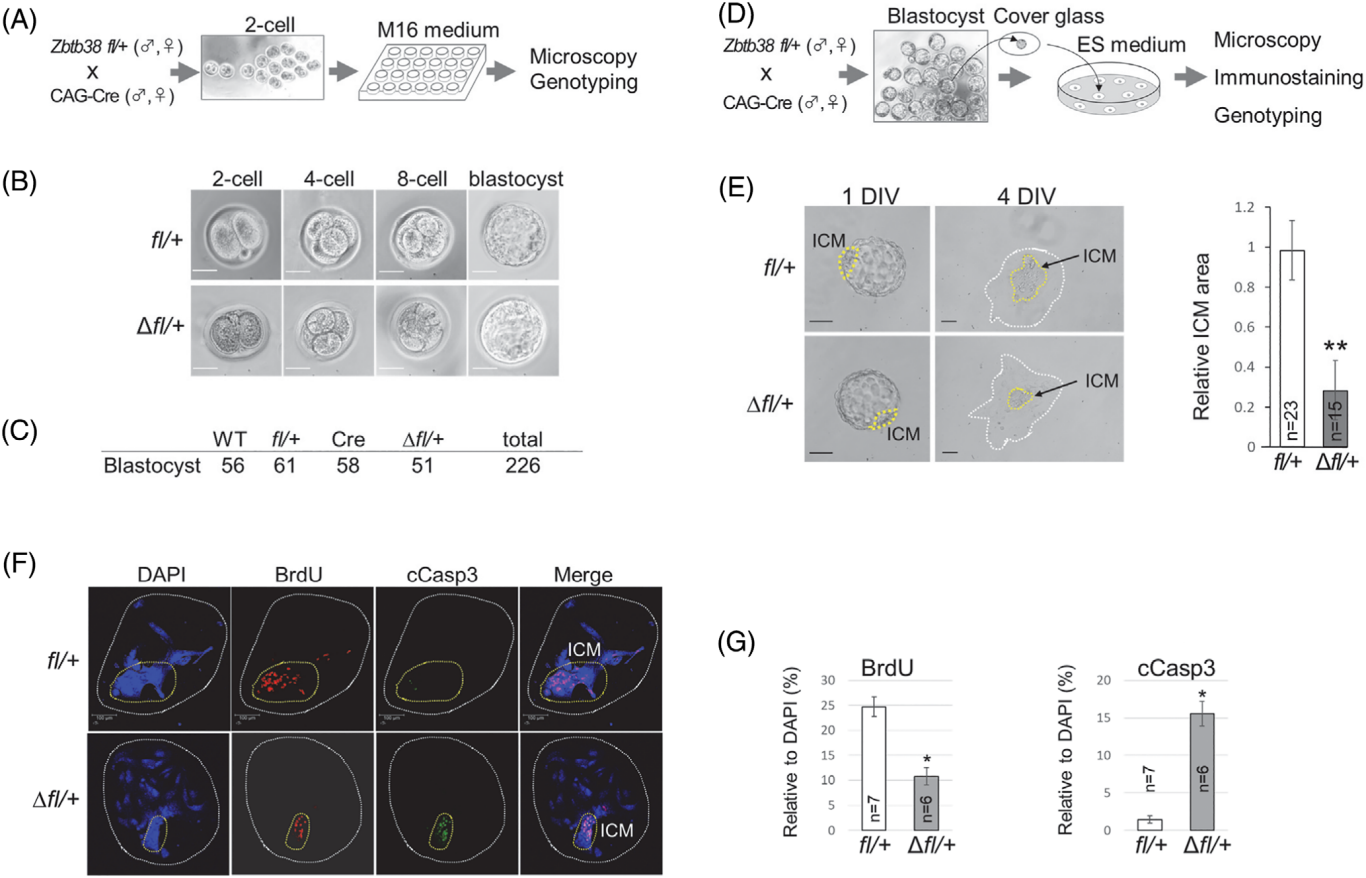
Effects of heterozygous loss of *Zbtb38* on pre‐ and peri‐implantation development in vitro. (A–C) Heterozygous loss of *Zbtb38* does not affect preimplantation development. (A) Illustration of the two‐cell embryo to the blastocyst in vitro culture. (B) Bright‐field microscopy of two‐cell embryo to the blastocyst stage. (C) Genotypes of blastocysts at day 4 were determined by PCR. (D–G) Heterozygous loss of *Zbtb38* suppresses inner cell mass (ICM) outgrowth. (D) Schematic diagram of embryo outgrowth in vitro. (E) Bright‐field microscopy of cultured blastocysts at 1 DIV and 4 DIV is shown (left panel). Yellow dashed lines denote ICM. Scale bar: 100 μm. Right panel, quantitative evaluation of relative ICM area (ICM/TE area). Images were analysed using ImageJ software. The data were representative of five independent experiments, and error bars indicate ± S.D. **p < 0.01. (F) Confocal immunofluorescence images of BrdU‐labelled and cCasp3‐positive cells of 4 DIV embryos. DNA was counterstained with DAPI. Yellow dashed lines denote ICM. Scale bar, 100 μm. (G) Quantitative analysis of the number of BrdU‐positive cells (left) or cCasp3‐positive cells (right) relative to the total number of nuclei (DAPI‐positive cells) from the indicated numbers of representative embryos. Error bars represent ± S.D. *p < 0.05

Because Zbtb38 is expressed from the two‐cell to blastocyst stages (Figure S7), we investigated whether Zbtb38 loss affects pre‐implantation development. To test this possibility, two‐cell embryos from the *Zbtb38 fl/+* and CAG‐Cre intercross were cultured in vitro until the four‐cell, eight‐cell, and blastocyst stages (Figure 5A,B). Embryos were monitored daily under a microscope, and blastocysts were subsequently analysed by immunohistochemistry staining and were genotyped. Of the 226 blastocysts assessed, an expected Mendelian ratio was observed among the four genotypes (Figure 5C). During development from the two‐cell to blastocyst stage, the *Zbtb38* Δ*fl/+* embryos appeared to be morphologically indistinguishable from those of their control embryos (Figure 5B). Moreover, proliferation and apoptosis were similar between the Δ*fl/+* and control blastocysts, as evidenced by the equivalent number of BrdU‐ and cCasp3‐positive cells (Figure S8A,B). These results suggest that the heterozygous loss of *Zbtb38* does not inhibit embryonic development during the pre‐implantation stage.

We next investigated whether loss of Zbtb38 affects blastocyst outgrowth, which recapitulates peri‐implantation development in vivo. For this purpose, blastocysts obtained from the *Zbtb38 fl/+* and CAG‐Cre intercross were isolated and cultured for 4 days in vitro (DIV) to support further embryonic development beyond the blastocyst stage (Figure 5D). Both *Zbtb38* Δ*fl/+* and control blastocysts normally hatched from zonae pellucidae, attached to the culture dish and initiated outgrowth at 2 DIV, and TE cells differentiated into largely polypoid trophoblast giant cells at 3–4 DIV with no visible difference (Figure 5E,F). Notably, although cells from the control ICM proliferated to expand the area, cells from the Δ*fl/+* ICM showed a dramatic proliferation defect with a markedly smaller proliferative zone (Figure 5E). To explore whether proliferation and apoptosis account for the aforementioned phenotypes, four DIV embryos were immunostained and analysed. The results showed that outgrowth of the control blastocysts displayed high proliferation and a low degree of apoptosis, particularly in ICM‐derived cells, as evidenced by the high BrdU incorporation with rare cCasp3‐positive cells in ICM (Figure 5F,G). In contrast, a decreased number of proliferating cells and an increased number of apoptotic cells were observed in *Zbtb38* Δ*fl/+* ICM. These data demonstrate that Zbtb38 expression is essential for peri‐implantation development.

### 3.5 Changes in gene expression in *Zbtb38^+/−^* embryos and *Zbtb38* ^*+/−*^ ES cells

**FIGURE 6:**
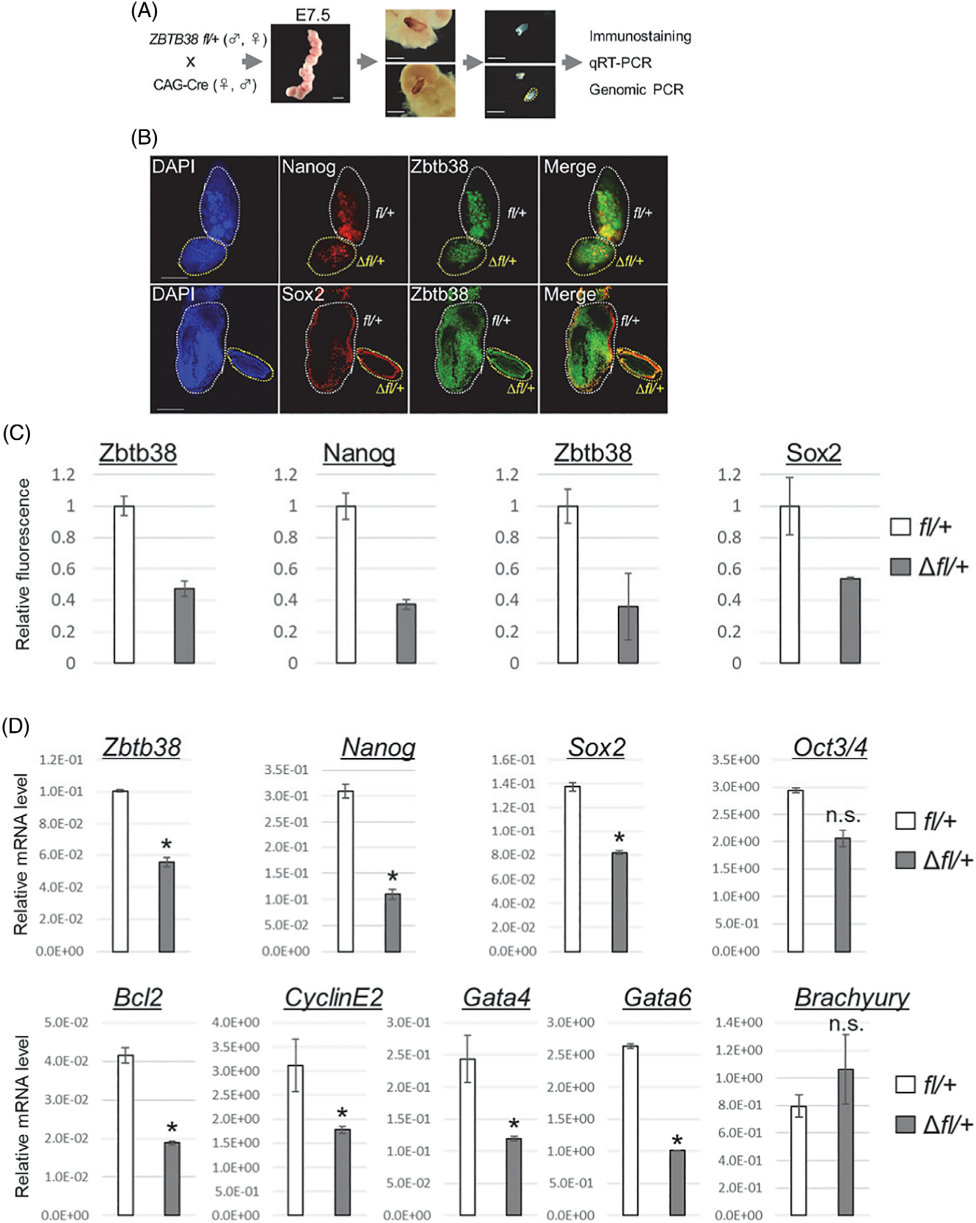
Expression levels of Zbtb38 and markers in E7.5 embryos. Loss of *Zbtb38* single allele results in a misregulation of pluripotency, proliferation, differentiation, and apoptotic genes in E7.5 embryos. (A) Schematic diagram of isolated E7.5 foetuses. (B and C) Confocal images of whole‐mount immunohistochemical staining (B) and relative fluorescent intensity (C). (B) Expressions of the indicated proteins were detected by anti‐Zbtb38, anti‐Nanog, and anti‐Sox2 antibodies. Cell nuclei were counterstained with DAPI. Scale bar denotes 50 μm. (C) Representative relative fluorescence as a measure of Zbtb38 and Nanog from the upper panel of (B) (*fl/+*: *n* = 2; ∆*fl/+*: *n* = 2), and a measure of Zbtb38 and Sox2 from the lower panel of (B) (*fl/+*: *n* = 2; ∆*fl/+*: *n* = 2). *fl/+* was normalized to 1, and error bars indicate ± S.D. (D) qRT‐PCR results of the indicated gene expressions of the *Zbtb38* ∆*fl/+* embryo and control embryo. Data shown are representative of three independent experiments, and error bars represent ± S.D. *p < 0.05

To investigate the molecular mechanism by which Zbtb38 loss leads to defective embryos, we focused on E7.5 embryos, which is the stage at which all *Zbtb38* ^*+/−*^ embryos were dramatically smaller than their controls but could be entirely isolated (Table 1 and Figure 3). For this purpose, we carefully isolated the E7.5 foetuses from the intercrosses of *Zbtb38 fl/+* and CAG‐Cre mice (Figure 6A). Whole‐mount immunofluorescence analysis showed that Zbtb38, Nanog and Sox2, albeit to a lesser extent, were downregulated in the *Zbtb38* Δ*fl/+* embryos as compared to their control littermates (Figure 6B,C). Moreover, the qRT‐PCR analysis showed that heterozygotic loss of *Zbtb38* reduced *Zbtb38* (dropped by 50%), *Nanog* (dropped by 64%), and *Sox2* (dropped by 40%) levels as compared with the controls (Figure 6D). mRNA levels of *Cyclin E2* and *Bcl2*, markers of G1/S transition and anti‐apoptosis, respectively, were downregulated in the *Zbtb38* Δ*fl/+* embryos as compared to their controls (Figure 6D). Furthermore, the ExE markers of *Gata4* and *Gata6* were downregulated, whereas the mesodermal gene, *Brachyury*, was not affected. Collectively, these findings demonstrate that loss of *Zbtb38* single allele results in a half decrease of Zbtb38 expression (mRNA and protein) in vivo, giving rise to misregulation of pluripotency, proliferation, differentiation and apoptotic genes.

**FIGURE 7:**
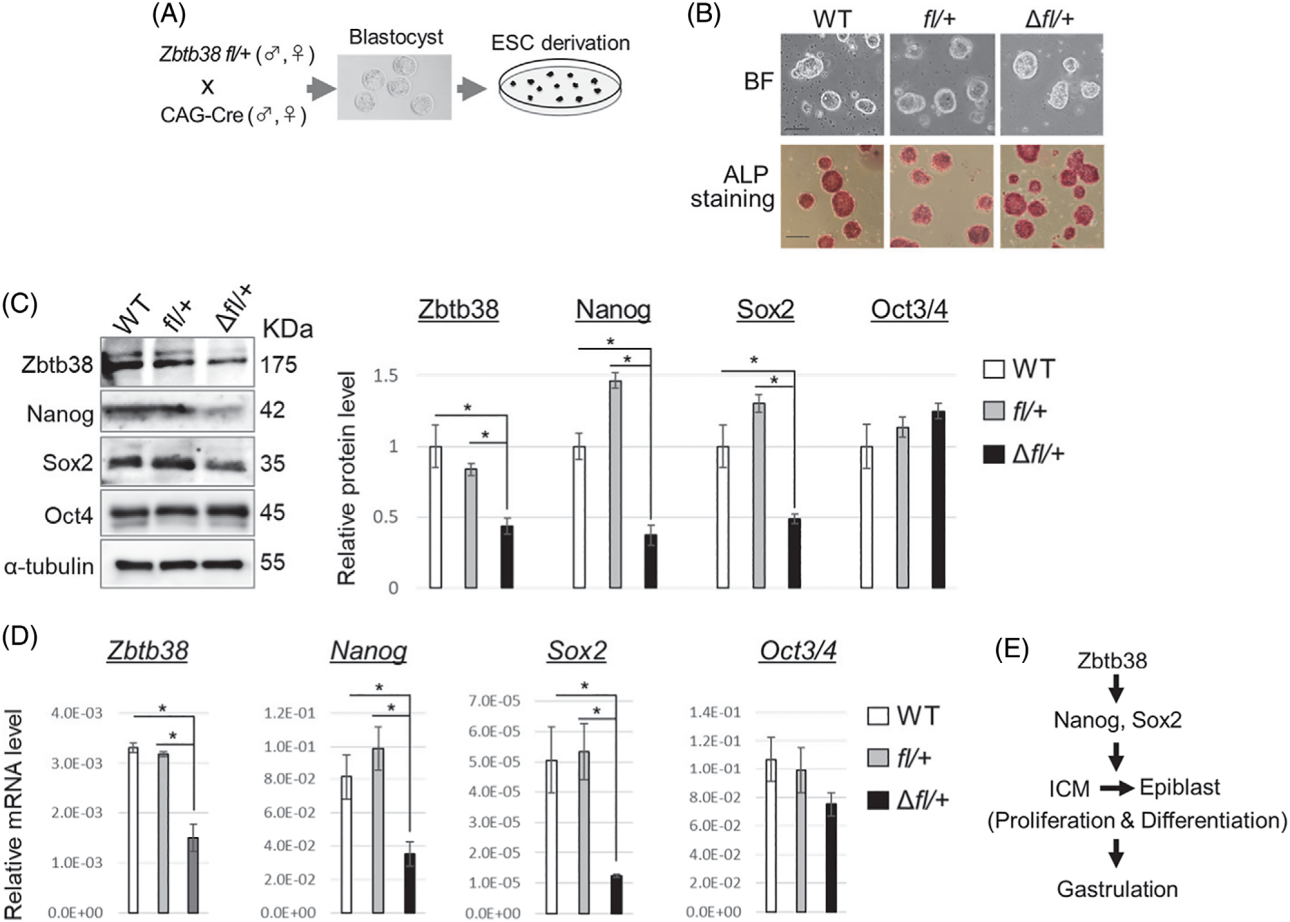
Establishment of embryonic stem (ES) cell clones and expressions of genes in ES cells. (A) Schematic diagram showing the derivation of ES cells‐ from blastocysts. (B) Bright‐field images of typical ES cell colonies (top) which show compact colonies with distinct borders and well‐defined edges, and alkaline phosphatase staining (bottom) of ES cells‐. Scale bar, 100 μm. (C and D) loss of a single *Zbtb38* allele in ES cells leads to a reduced expression of Nanog, Sox2 in ES cells. Expressions of the indicated proten‐ (C) and ‐mRNA in (D) from ES cells. *GAPDH* and α‐tubulin were used as internal controls for qRT‐PCR and immunoblotting, respectively. Data shown are representative of three independent experiments, and error bars indicate ± S.D. *p < 0.05. (E) Schematic model of how Zbtb38 affects gastrulation

To investigate the effects of Zbtb38 loss on ES cell pluripotency and self‐renewal, blastocysts from the *Zbtb38 fl/+* and CAG‐Cre intercross were cultured in ES medium to generate ES cells (Figure 7A). ES colonies derived from WT, *Zbtb38 fl/+*, and Δ*fl/+* blastocyst cultures were established without differences in frequencies (data not shown), indicating that Zbtb38 is dispensable for the generation of ES cells. We found that the *Zbtb38* ^*+/−*^ ES cells retained their morphological features of undifferentiated ES cells, stained similarly positive for alkaline phosphatase activity, indicative of the pluripotent, undifferentiated state (Figure 7B). To further characterize these ES cell properties, the expression of pluripotent genes was examined by western blotting and qRT‐PCR. As shown in Figure 7C,D, Zbtb38 mRNA and protein levels in the Δ*fl/+* ES cells were reduced to half as compared to those of their controls, indicating that the loss of a single *Zbtb38* allele led to a half decrease in its expression in vitro. Moreover, the mRNA and protein levels of Nanog and Sox2, except those of Oct4, were downregulated in the *Zbtb38* ^*+/−*^ ES cells as compared to those in the controls (Figure 7C,D).

Altogether, these findings demonstrate that heterozygous loss of *Zbtb38* did not affect pre‐implantation development, but instead suppressed ICM outgrowth during peri‐implantation. Heterozygous loss of *Zbtb38* results in the downregulation of Nanog and Sox2, and this decrease leads to hypo‐proliferation and apoptosis in the epiblasts, resulting in gastrulation defects (Figure 7E).

## 4 DISCUSSION

Currently, the physiological necessity of MBP during peri‐implantation remains almost unknown, although Dnmts play critical roles in this stage. In agreement with the developmental phenotypes of Dnmts KO mice,^16^, ^17^ our results showed that *Zbtb38* ^*+/−*^ embryos also died around E9.5, indicating that Zbtb38 is essential for peri‐implantation development. This finding demonstrates that a methyl‐CpG binding protein finally has an embryonic phenotype, unlike MeCP2, Mbd2, Mbd1 and Zbtb33. Because of the complexity of methylation pattern dynamics and technical limitations at the early embryogenesis, where, when, and how Zbtb38 binds to the methyl‐CpG sites of genes, transposons, and genome, remain largely unknown. Further experiments using methyl‐ChIP‐seq are required to explore the Zbtb38 binding dynamics and properties in ES cells and early embryos (WT vs. heterozygote).

In this study, both the conventional and Cre‐loxP‐based conditional knockout approaches revealed that loss of the *Zbtb38* single allele resulted in embryonic lethality. To our knowledge, only two of the known 6014 genes (0.03%) KO databases (https://www.mousephenotype.org/), the vascular endothelial growth factor (VEGF) and the Notch ligand DLL4, have been shown to exhibit heterozygous embryonic lethality. The *VEGF* ^*+/−*^ and *DLL4* ^*+/−*^ mice, generated by a conventional targeting strategy, died around E10.5 because of vascular developmental failure.^48^, ^49^ However, these results were reported 20 to 30 years ago, a half decrease of the VEGF and DLL4 genes in heterozygous KO embryos or ES cells has not been demonstrated, probably due to technical constraints. Our data indicate that *Zbtb38* mRNA levels in the heterozygous embryos and ES cells, and Zbtb38 protein levels in the heterozygous ES cells decreased to half that of the controls. In support of this notion, loss of Zbtb38 altered the expression of genes critical for pluripotency, proliferation, differentiation and apoptosis. Our findings provide new insights for the understanding of a very low percentage of heterozygous embryonic lethality.

Wong et al. generated *Zbtb38* ^*−/−*^ mice using CRISPR/Cas9 technology in a C57BL/6 background.^50^ The *Zbtb38 fl/fl* mice were crossed with CMV‐Cre mice expressing Cre recombinase ubiquitously under the control of a human cytomegalovirus promoter to obtain germline *Zbtb38* deletion. Unexpectedly, *Zbtb38* ^*−/−*^ mice were born, developed normally, and were fertile, with no detectable abnormalities of massive gene expression. However, expression levels of either the mRNA or protein of Zbtb38 was not demonstrated in the tales of their constitutive KO mice, leaving open the possibility that the normal phenotype observed is not the consequence of the mice lacking Zbtb38.

The results presented in this study indicate that Zbtb38 gene haploinsufficiency downregulated *Nanog* and *Sox2* expression. It has been shown that either the *Nanog* ^*−/−*^ or *Sox2* ^*−/−*^ mice died soon after implantation, whereas their heterozygous mice appeared normally and were fertile,^36^, ^51^ suggesting that their dosage is critical for embryogenesis. Based on the following findings, we concluded that the loss of Nanog and Sox2 expression accounts, at least partially, for the phenotypic outcome of *Zbtb38* ^*+/−*^ embryos. First, Zbtb38 largely colocalized with Nanog and Sox2 in the ICM of blastocysts, and partially colocalized with them in the epiblast of E6.5 embryos. Second, immunofluorescence data showed that Nanog and Sox2 were downregulated in the E7.5 *Zbtb38* ^*+/−*^ embryos. Third, qRT‐PCR results revealed that heterozygous loss of *Zbtb38* led to reduction of *Nanog* and *Sox2* expression in E7.5 embryo and in ICM‐derived ES cells. Fourth, our previous data have revealed that Zbtb38 positively regulates ES cell (RF8, derived from 129/TerSv mice) proliferation by regulating its downstream target Nanog, and the phosphoinositide 3‐kinase (PI3K) signalling pathway accounts for this regulation.^30^ Notably, the loss of Zbtb38 (knockdown, heterozygous and homozygous KO) inhibited ES cell proliferation via downregulation of Nanog expression, whereas the constitutive overexpression of Nanog rescued the proliferation defect caused by *Zbtb38* knockdown.^30^

To date, the detailed molecular mechanism by which Zbtb38 regulates *Nanog* and *Sox2* expression remains to be elucidated. In ES cells, Nanog activates *Sox2* transcription, and vice versa. In embryos, however, Nanog is not required to initiate transcription of *Sox2*, and vice versa. Both the *Nanog* promoter and the *Sox2* enhancer are largely unmethylated in blastocysts and ES cells‐, but they are methylated in differentiated cells,^52^, ^53^ suggesting that DNA methylation is linked to their expression state. In support of this notion, the consensus binding sites (the methylated CGCCAT^54^ and non‐methylated CAGGTG, unpublished data) for Zbtb38 were found in the *Nanog* promoter and *Sox2* enhancer, and biochemical experiments are undertaken to explore how Zbtb38 regulates their expressions. In addition, we cannot exclude the possibility that Zbtb38 may recruit unknown factor(s), which, in turn, modulates *Nanog* and *Sox2* expression.

Noticeably, transcriptional factors play critical roles in maintaining ES cell undifferentiation, whereas the repressive epigenetic chromatin plays a minor role.^55^ This epigenetic paradox of ES cells tolerates a loss of many epigenetic factors and does not affect the transition of ES cells into ground state pluripotency.^56^ Consistently, our data in this study and our previous report showed that undifferentiated ES cells remain largely unaffected by *Zbtb38* depletion.^30^ After implantation, however, DNA methylation is dispensable for the ExE development while is required for the epiblast differentiation.^57^ The requirement of DNA methylation for the ExE lineage but not for the epiblast lineage, is consistent with our data that loss of Zbtb38 suppresses epiblast proliferation.

Zbtb38 is ubiquitously expressed in tissues,^28^ and its expression is associated with height,^37^ cancers,^38^, ^39^, ^40^, ^58^ neurodegenerative diseases,^41^, ^42^ rheumatoid arthritis.^59^ Moreover, down‐regulation of ZBTB38 expression potentiates the toxicity of anti‐tumour reagents in cancer cells.^60^ Thus, the generation and analysis of the tissue‐specific Cre‐mediated KO (hetero and homo) mice will comprehend Zbtb38's physiological functions and Zbtb38‐associated diseases.

In conclusion, our data showed that germline loss of the *Zbtb38* single allele decreased epiblast cell proliferation and increased apoptosis shortly after implantation, leading to early embryonic lethality. Our results demonstrated that the phenotypes caused by heterozygous loss of *Zbtb38* depend, at least partially, on the suppression of *Nanog* and *Sox2* expressions. As *Zbtb38* expression increases during embryonic development, the generation of time‐dependent conditional *Zbtb38* KO mice will allow us to understand its physiological function in organogenesis during mid‐to‐late‐stage embryonic development.

## CONFLICT OF INTEREST

The authors declare no conflict of interest exists.

## AUTHOR CONTRIBUTIONS

Miki Nishio and Takuya Matsuura were involved in the conception and design of the study, performed the experiments, data analyses and collected and assembled data. Shunya Hibi and Shiomi Ohta conducted the experiments; Chio Oka, Noriaki Sasai and Yasumasa Ishida offered the assistance of the animal experiments, technical guidance and advice; Eishou Matsuda was involved in the conception, design, performed the experiments, data analyses, and was responsible for manuscript writing and final approval of the manuscript. All authors read and approved the manuscript.

## Supporting information


**Appendix S1**: Supporting Information

## Data Availability

The data used to support the findings of this study are available from the corresponding author upon reasonable request.
