# Supplementary material for: Heterozygous loss of Zbtb38 leads to early embryonic lethality via the suppression of Nanog and Sox2 expression
Source: Cell Prolif. 2022 Mar 17;55(4):e13215. doi: 10.1111/cpr.13215 (PMC9055898; doi:10.1111/cpr.13215)
Supplement: Supplementary file 1 — Appendix S1: Supporting Information [file CPR-55-e13215-s001.docx]

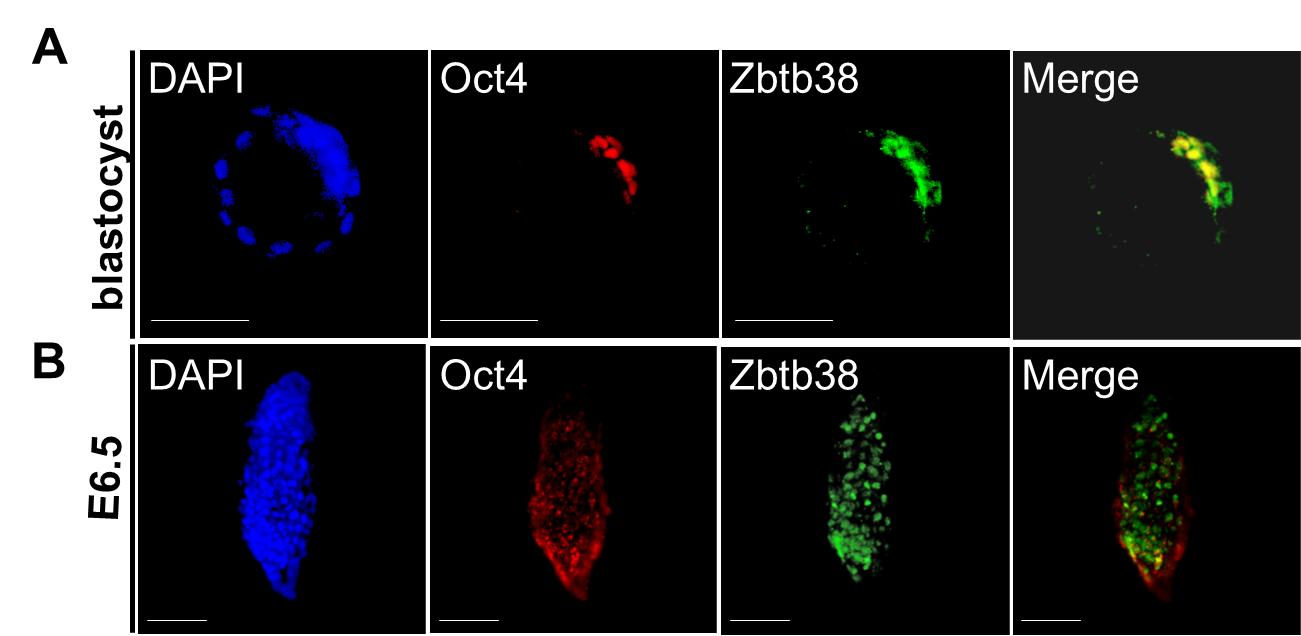

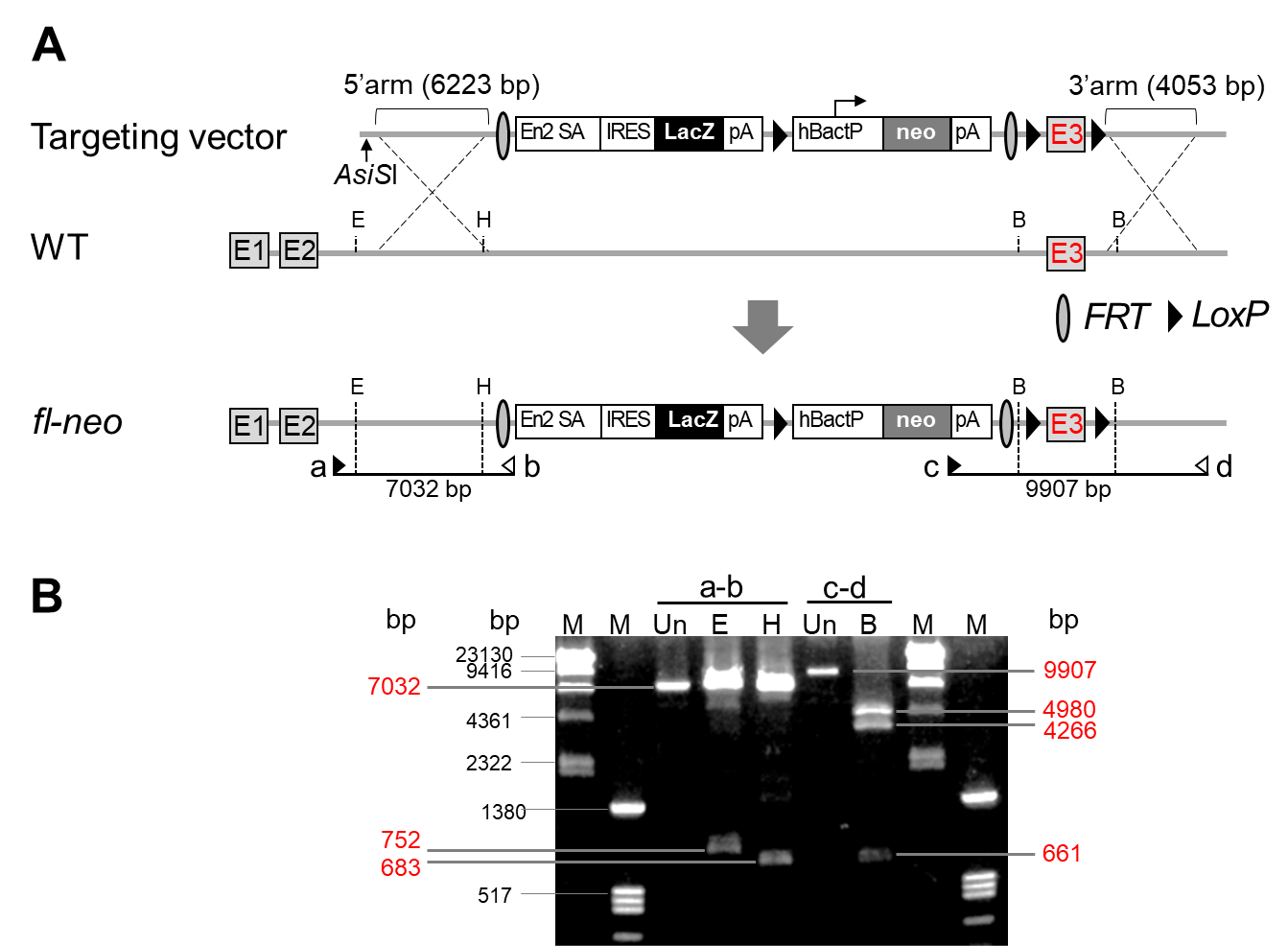


**Figure S1. Expression patterns of Zbtb38**

**(A)** Whole-mount immunofluorescence and confocal microscopy for blastocyst (A) and E6.5 **(B)**. Expression patterns of the indicated proteins were detected by anti-Zbtb38 and anti-Oct4 antibodies. Cell nuclei was counterstained with DAPI. Scale bar denotes 50 μm (A) or 100 μm (B). ExE: extraembryonic ectoderm, epi: epiblast.

**Figure S2. Schematic representation of the targeting vector and locus**

**(A)** Schematic of EUCOMM targeting vector. The vector contains 10kb of *Zbtb38* homologous sequence in which the 5′ and 3′ arms of homology are 6.2 kb and 4.1 kb, respectively. Homologous recombination in ES cells results in the generation of the *fl-neo* targeted allele. Abbreviations, SA: splice acceptor, IRES: internal ribosome entry site, pA: poly(A) signal, En2 SA: mouse En2 splicing acceptor, IRES: internal ribosome entry site, hBactP: human b-actin promoter. Primers (a-d) are shown by arrows and the expected size (bp) of genomic PCR are shown under the individual primer pairs. **(B)** Results of genomic PCR for confirmation of homologous recombination. The red number denotes the expected size of the bands digested with the indicated restrictive enzymes. E: *EcoR*I, B: *BamH*I, H:*Hind*III, Un: uncut. M denotes a size marker.


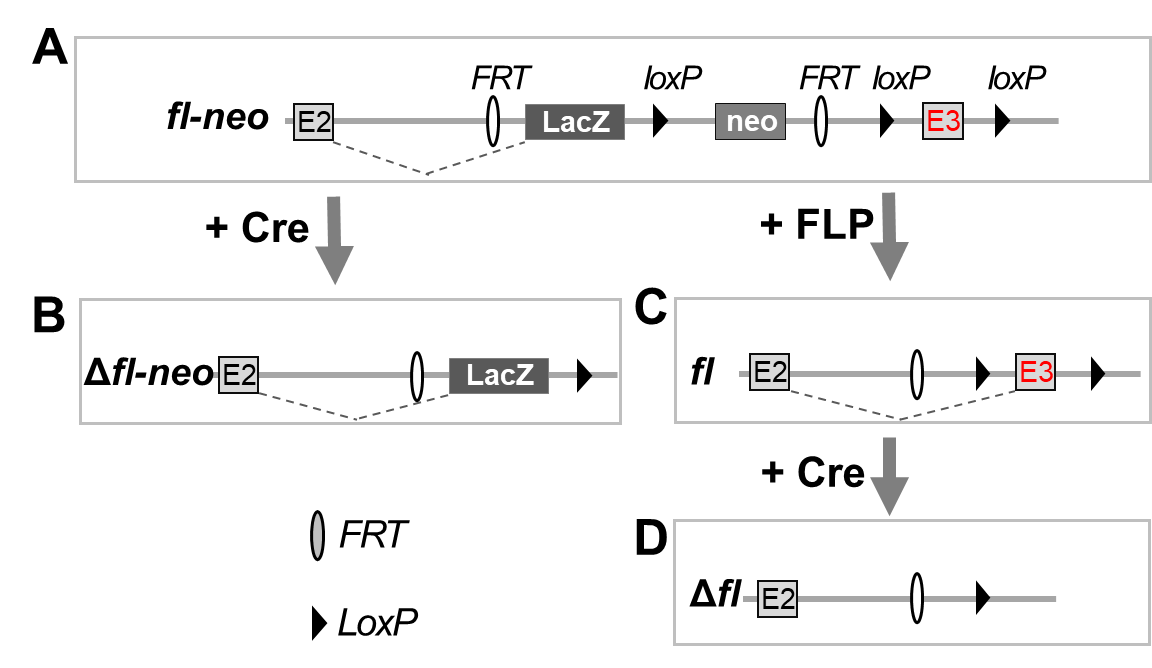


**Figure S3. Two strategies to generate *Zbtb38^+/-^* allele**

**(A)** *fl-neo* targeted allele. **(B)** Crossing the *fl-neo* allele to a mouse strain expressing CAG-Cre recombinase, yields a lacZ-tagged allele lacking *Zbtb38* exon 3 and neomycin cassette (∆fl-neo). **(C)** Crossing the *fl-neo* allele to a mouse line expressing FLP recombinase results in a conditional-ready allele lacking both the lacZ and neomycin cassettes (*fl*). **(D)** Crossing the *fl* allele a mouse strain expressing CAG-Cre recombinase leads to a null allele lacking *Zbtb38* exon 3 (*∆fl*). Exons are shown as empty boxes and marked by a number inside. Neo: neomycin-resistant gene; FLP: flp recombinase.


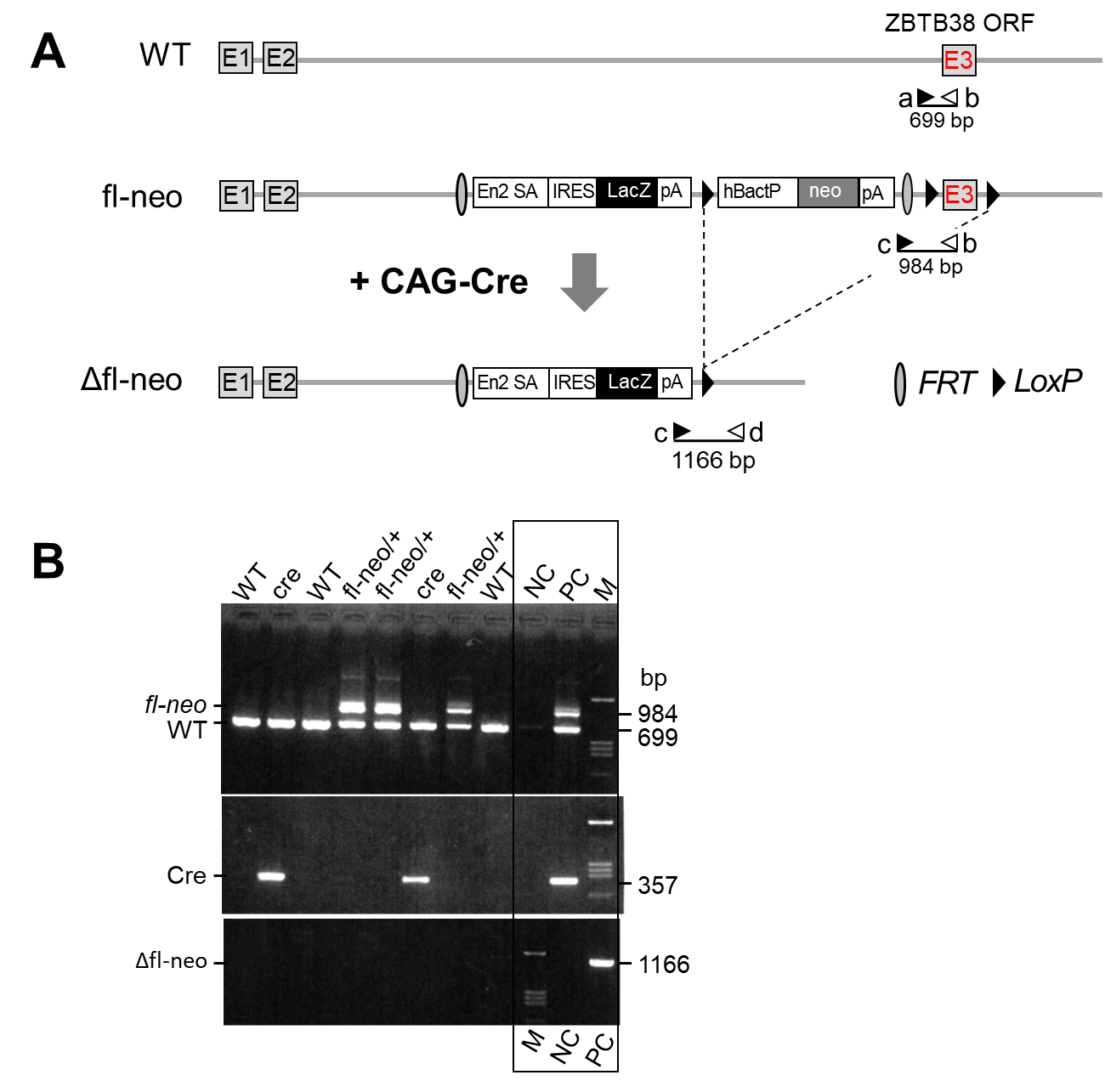


**Figure S4. Generation of the *Zbtb38* ∆*fl-neo/+* mice**

**(A)** Schematic representation of WT and knockout constructs. The deleted allele (∆*fl-neo*) lacking the *Zbtb38* exon 3 is produced by CAG-Cre-induced recombination as shown. The position of the primers (a~d) used for genotyping is shown with arrows, the expected size (bp) of genomic PCR are shown under the individual primer pairs. (B) PCR genotyping of the neonates from the Zbtb38 *fl-neo/+* mice and CAG-Cre mice intercrossed. WT, *fl-neo* and *∆fl-neo* alleles produced a 699-bp, 984-bp and 1166-bp band, respectively. Cre genotyping primer produces a 357 bp band. M: size marker; NC (negative control): ddw; PC (positive control): *fl-neo/+* ES cells and *∆fl-neo/+* ES cells were used for *fl-neo/+* (upper) and *∆fl-neo/+* (middle), CAG-Cre mice ear was used for Cre (lower).


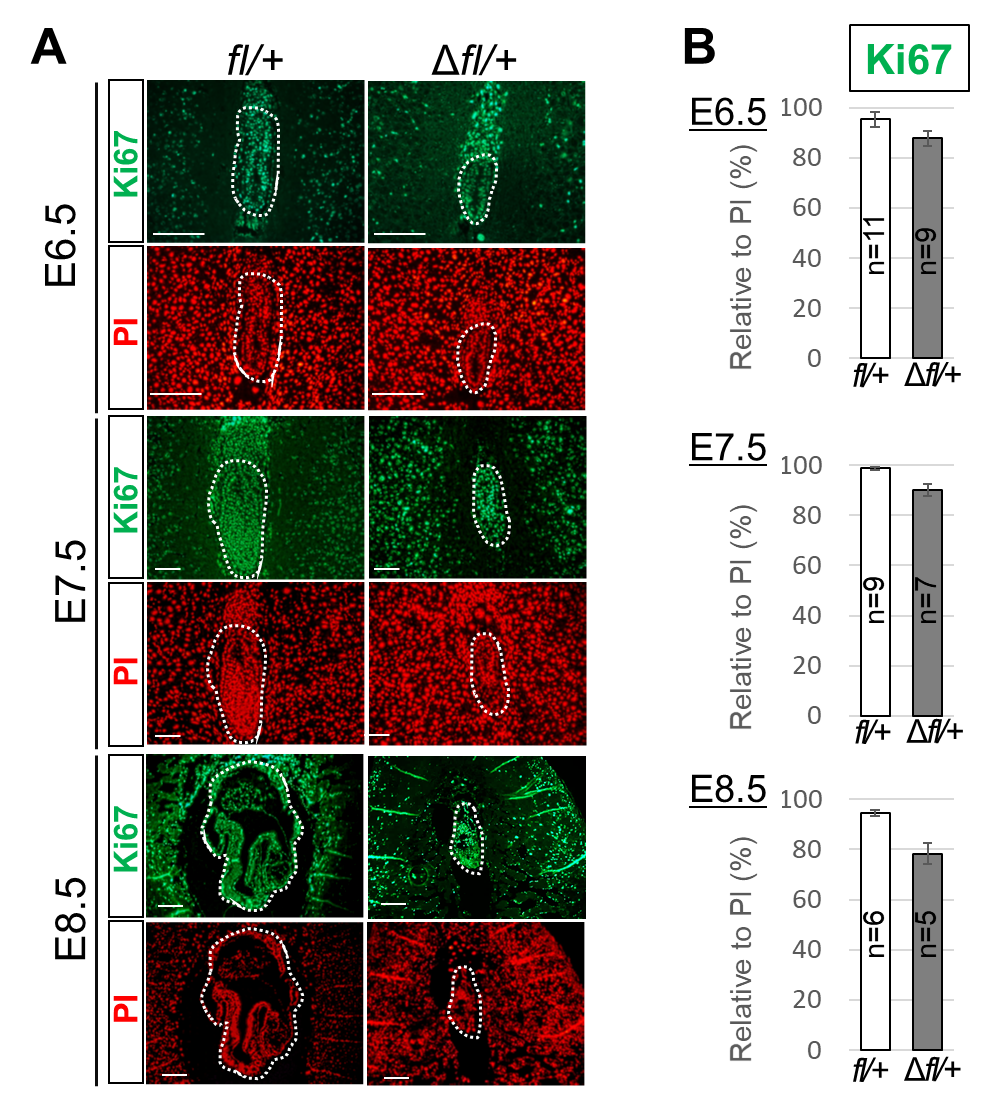


**Figure S5. Evaluation of proliferating cells in embryos**

**(A)** The number of Ki67-positive cells in paraffin-embedded sagittal sections of E6.5-E8.5 embryos. Immunofluorescence analysis of control *Zbtb38 fl/+* (a1~a6) and *Zbtb38* ∆*fl/+* (b1~b6) embryos. Paraffin-embedded sections were taken for performing immunostaining with anti-Ki67 antibody (green) and nuclei were counterstained with PI (red). Scale Bar: 50 μm. **(B)** Quantitative analysis of the number of labeled Ki67 cells relative to the total number of nuclei (PI-positive cells) from the indicated numbers of representative sections. Error bars represent ± S.E.M.


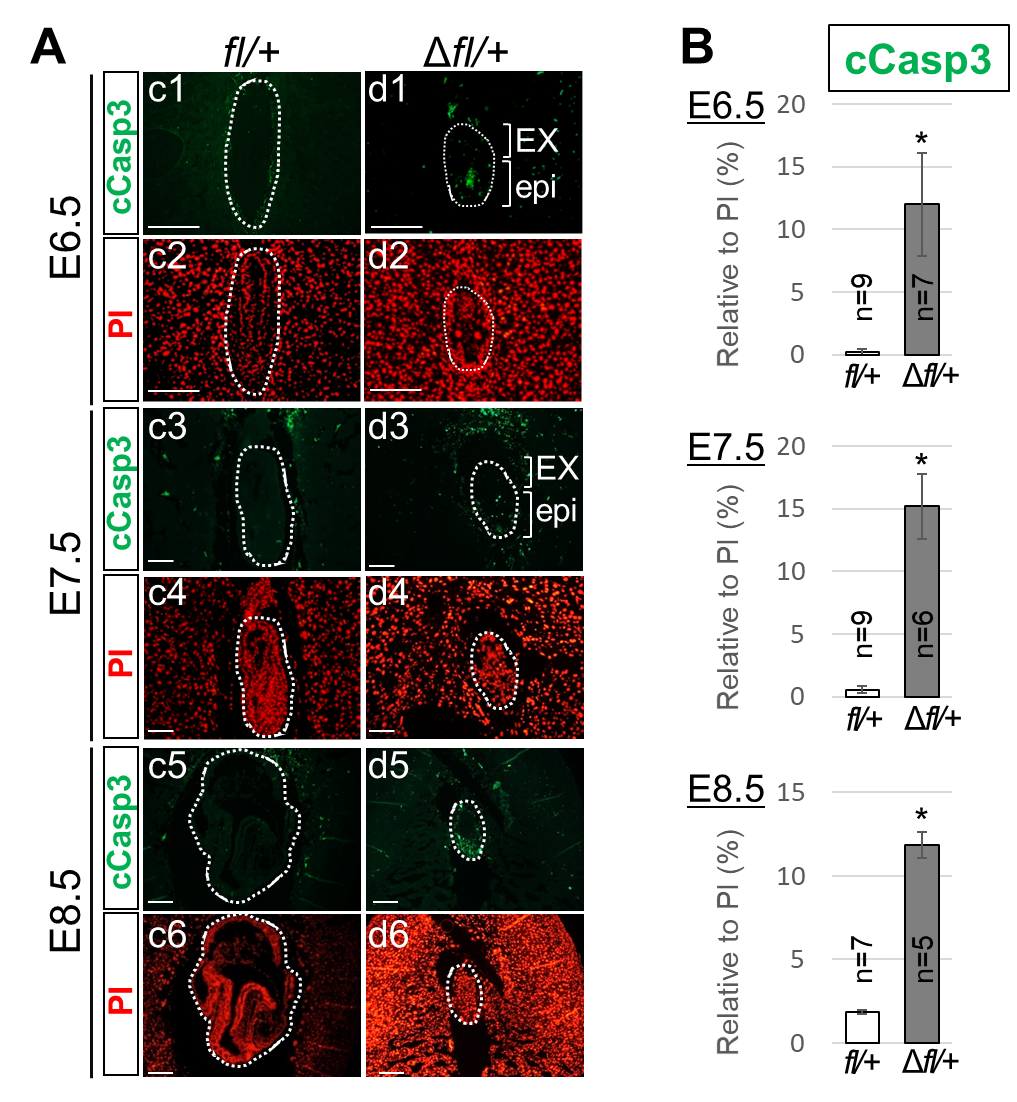


**Figure S6. Evaluation of apoptotic cells in embryos**

**(A)** Immunofluorescence assay was performed on paraffin-embedded sagittal sections from E6.5 onwards. cCasp3-positive cells are shown in green and nuclei were counterstained with PI (red). Scale Bar: 50 μm. **(B)** Quantitative analysis of the number of cCasp3-positive cells relative to the total number of nuclei (PI-positive cells) from the indicated numbers of representative sections. Error bars show ± S.E.M. **P* < 0.05.


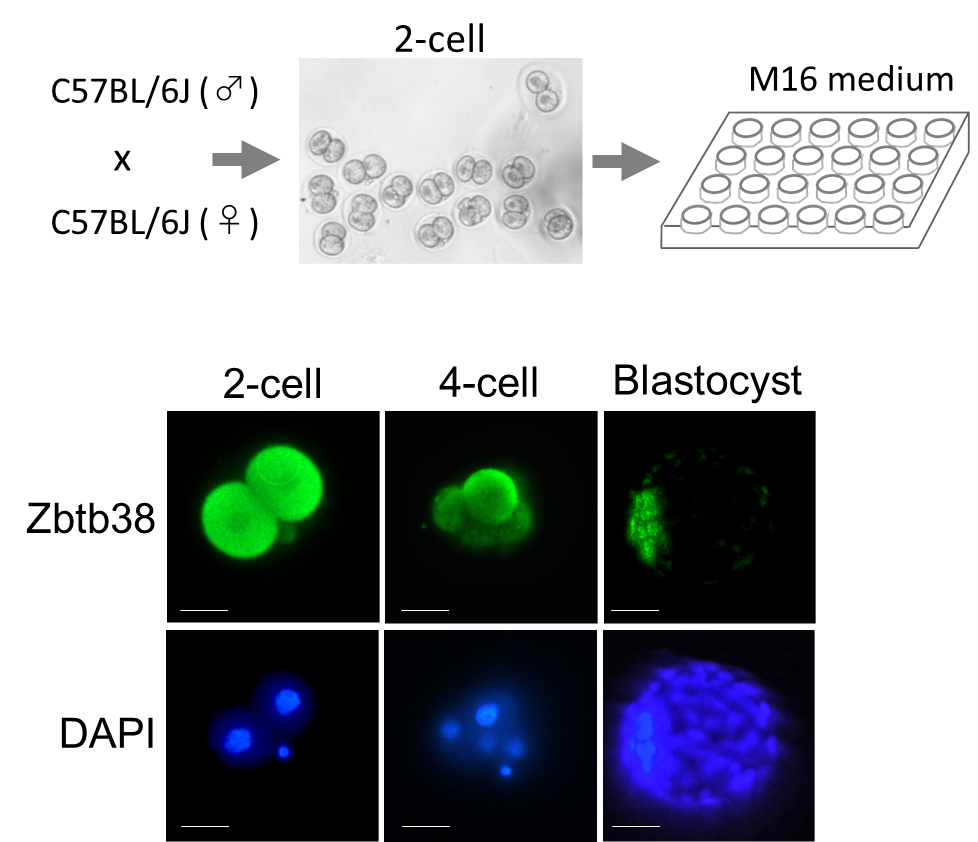


**Figure S7. Expression patterns of Zbtb38 in pre-implantation embryos**

Illustration of culturing 2-cell embryos to blastocysts *in vitro* (upper panel). Lower panel, immunofluorescence microscopy for Zbtb38 expression with anti-Zbtb38 antibody at the indicated stage of embryos. Cell nuclei were counterstained with DAPI. The scale bar denotes 50 μm.


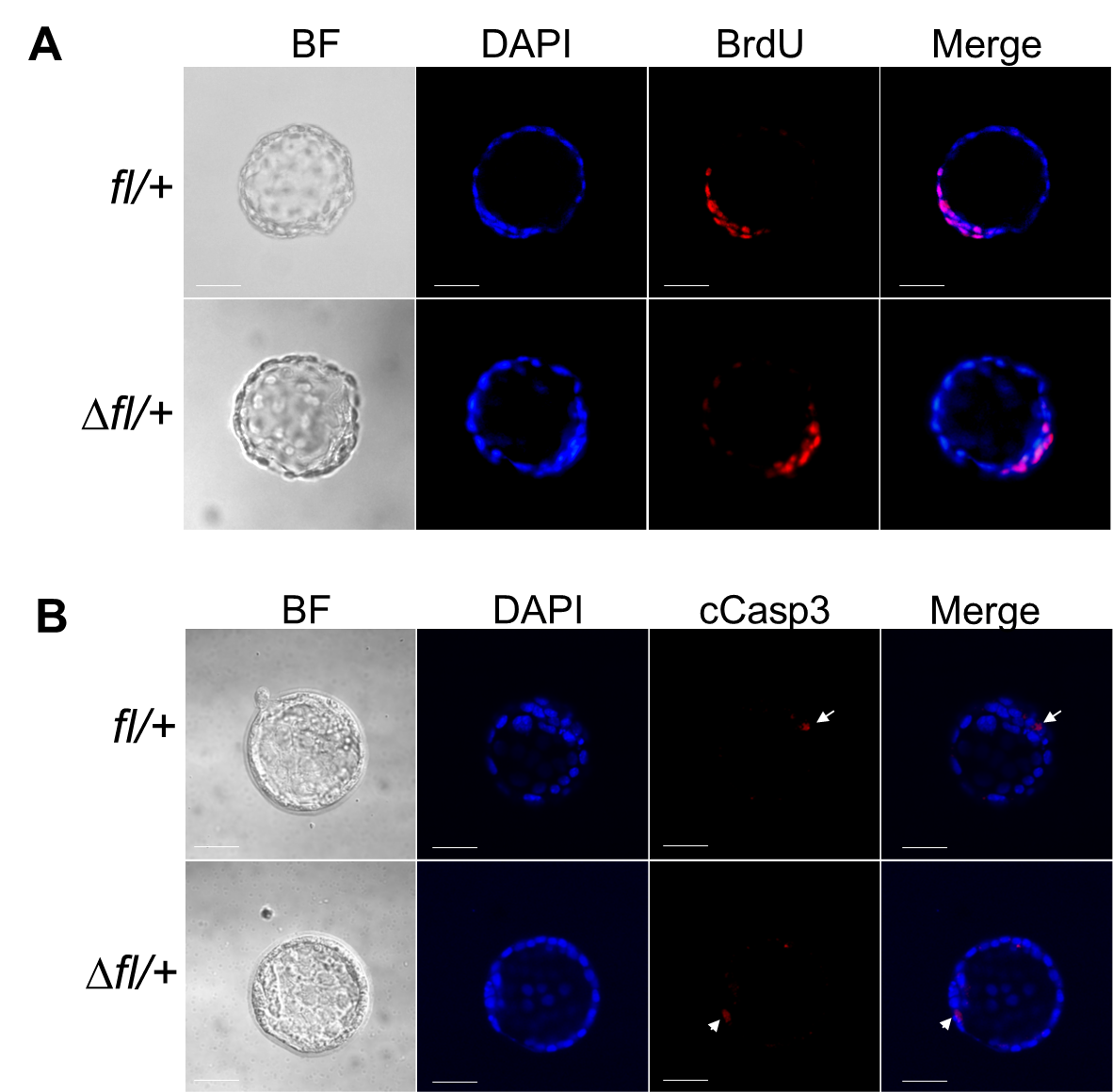


**Figure S8. Evaluation of proliferation and apoptosis in blastocyst**

**(A, B)** Whole-mount immunofluorescence and confocal microscopy of blastocysts from the *Zbtb38 fl/+* mice and CAG-Cre mice intercrossed. Immunostaining with anti-BrdU antibody (red, A) and anti-cCasp3 antibody (red, B) are shown. Arrows indicate cCasp3-positive cells. Nuclei were counterstained with DAPI (green). Scale bar, 50 μm.

**Supplemental Table S1.**

Genotype analysis of offspring from the *Zbtb38 fl-neo/+* and CAG-Cre intercrosses

| **stage** | **Total** | **WT** | ***fl-neo/+*** | **Cre** | **∆*fl-neo/+*** |
| --- | --- | --- | --- | --- | --- |
| **Newborn** | **96** | **37** | **30** | **29** | **0** |
| **E10.5** | **35** | **12** | **12** | **11** | **0** |
| **E9.5** | **30** | **6** | **14** | **7** | **3 (^#^3)** |
| **E8.5** | **31** | **7** | **9** | **8** | **7 (^*^7)** |

^#^absorbed; ^*^abnormal

**Supplemental Table S2.** Primer used for qRT-PCR

| Gene | Primer sequence (5′ to 3′) | Product size |
| --- | --- | --- |
| *ZBTB38* | F: ACACCCTTTCTGCTTGGAGATAC  R: GACTGTCATCTCAGAACACGCC | 185 bp |
| *Nanog* | F: TTCAGAAATCCCTTCCCTCG  R: AGTAGCAGACCCTTGTAAGC | 162 bp |
| *Sox2* | F: CCCACCTACAGCATGTCCTAC  R: GCCTCGGACTTGACCACAG | 83 bp |
| *Oct4* | F: TCACTCACATCGCCAATCAG  R: CCTGTAGCCTCATACTCTTCTC | 275 bp |
| *Bcl2* | F: CTTCGCAGAGATGTCCAGTC  R: AGGGCGATGTTGTCCACCAG | 192 bp |
| *CyclinE2* | F: CAGACTCTCCGCAAGAAAC  R: GCTGATTCCTCCAGACAGTA | 149 bp |
| *Gata4* | F: GTGAGCCTGTATGTAATGCC  R: CTGTGCCCATAGTGAGATGAC | 273 bp |
| *Gata6* | F: GGGAGAAACTGTGACAATGAC  R: ACGAACGCTTGTGAAATGTG | 165 bp |
| *Brachyury* | F: GCTTCAAGGAGCTAACTAACGAG  R: CCAGCAAGAAAGAGTACATGGC | 117 bp |
| *TBP* | F: CTACCGTGAATCTTGGCTGTAAAC  R: AATCAACGCAGTTGTCCGTGGC | 121 bp |
| *GAPDH* | F: CAATGTGTCCGTCGTGGATCT  R: GTCCTCAGTGTAGCCCAAGATG | 124 bp |

**Supplemental Table S3.**

| **Primers for genomic PCR of Figure 2A and B** |
| --- |
| a: TGCTTGGAGATACAGAAACTTGCCCTAC |
| b: CCTGGAGAGGGACATGACTGTC |
| c: ACACCCTTTCTGCTTGGAGATAC |
| d: GATACTAGGGTCGTTAGAGGATTCAGC |
| Cre-F: ATGTCCAATTTACTGACCG |
| Cre-R: CGCCGCATAACCAGTGAAAC |
| **Primers for genomic PCR of Figure S4** |
| a: TGCTTGGAGATACAGAAACTTGCCCTAC |
| b: TTTGAGAAGTTGCGGTCACTAAGGTC |
| c: CACACCTCCCCCTGAACCTGAAAC |
| d: CTCAACCTCCTGCGTGCTGAT |
| Cre-F: ATGTCCAATTTACTGACCG |
| Cre-R: CGCCGCATAACCAGTGAAAC |
